# Effect of leisure-time physical activity on blood pressure in people with hypertension: a systematic review and meta-analysis

**DOI:** 10.1038/s41598-023-37149-2

**Published:** 2023-06-30

**Authors:** Md Shariful Islam, Ammatul Fardousi, Monaemul Islam Sizear, Md. Golam Rabbani, Rubana Islam, K. M. Saif-Ur-Rahman

**Affiliations:** 1 Infectious Diseases Division, icddr,b, Dhaka, Bangladesh; 2 Health Systems and Population Studies Division, icddr,b, Dhaka, Bangladesh; 3 Health Systems for Tuberculosis, Dhaka, Bangladesh; 4 Public Health Foundation, Bangladesh, Dhaka, Bangladesh; 5 Health Economics Unit, Health Services Division, Ministry of Health and Family Welfare, Dhaka, Bangladesh; 6 Independent researcher, Dhaka, Bangladesh

**Keywords:** Public health, Lifestyle modification

## Abstract

High blood pressure is a major risk factor for premature death. Leisure-time physical activities have been recommended to control hypertension. Studies examining how leisure-time physical activity affects blood pressure have found mixed results. We aimed to conduct a systematic review examining the effect of leisure-time physical activity (LTPA) on lowering blood pressure among adults living with hypertension. We searched studies in Embase, Medline/PubMed, Web of Science, Physical Education Index, Scopus and CENTRAL (the Cochrane Library). The primary outcome variables were systolic blood pressure (SBP) and diastolic blood pressure (DBP). This systematic review is registered on PROSPERO (CRD42021260751). We included 17 studies out of 12,046 screened articles in this review. Moderate-intensity LTPA (all types) reduced SBP compared to the non-intervention control group (MD −5.35 mm Hg, 95% CI −8.06 to −2.65, nine trials, n = 531, low certainty of the evidence). Mean DBP was reduced by −4.76 mm Hg (95% CI −8.35 to −1.17, nine trials, n = 531, low certainty of the evidence) in all types of LTPA (moderate intensity) group compared to the non-intervention control group. Leisure-time walking reduced mean SBP by −8.36 mmHg, 95% CI −13.39 to −3.32, three trials, n = 128, low certainty of the evidence). Walking during leisure time reduced −5.03 mmHg mean DBP, 95% CI −8.23 to −1.84, three trials, n = 128, low certainty of the evidence). Performing physical activity during free time probably reduces SBP and DBP (low certainty of the evidence) among adults with hypertension.

## Introduction

Hypertension is a leading risk factor for premature death worldwide^1,2^. Thresholds of hypertension differed between American and European guidelines. The threshold value of hypertension according to American guidelines is 130/80 mmHg^3^, whereas European guidelines defined hypertension when blood pressure is greater than 140/90 mmHg^4^. High systolic blood pressure (according to American guidelines) was responsible for 10.8 million deaths which was 19% of total deaths globally in 2019^2^. Globally, 1.3 billion people aged 30–79 years old lived with hypertension in 2019 (according to European guidelines). More than 1 billion people worldwide (82% of those with hypertension) live in low and middle-income countries^5^. Hypertension will affect 1.60 billion people worldwide by 2025^6^. Hypertension has imposed a substantial economic burden, including direct healthcare expenses ($370 billion/year)^7^ as well as indirect costs associated with lost productivity, premature death, and morbidity^8,9^.

The reduction of every 10 mm Hg in systolic blood pressure (SBP) and 5 mm Hg in diastolic blood pressure (DBP) could reduce cardiovascular events by a quarter, stroke by a third, and all-cause mortality by 13%^10–12^. Even, a 2 mm SBP drop can have clinically significant impacts on mortality^10–13^. People with lower heart rates (70 to < 72 beats/min) show a 50% reduced cardiovascular-related heart mortality and heart failure compared to those with higher heart rates (≥ 87 beats/min)^14^. Pharmacological treatment is proven to significantly improve the primary prevention of cardiovascular disease mortality and morbidity^12,15,16^. However, in a pooled analysis of 104 million participants, only 40% of people with hypertension received pharmacological treatment, and 23% of women and 18% of men of the total participants had controlled blood pressure^5^. Also, poor drug adherence is a major issue to manage hypertension^17^. Given the above drawbacks to drug adherence, exercise, low salt intake, quitting smoking, and controlling overweight are non-pharmaceutical therapies that can play a substantial role cost-effectively in managing hypertension^18–22^.

World Health Organization defines physical activity as “any movement of the body involving the skeletal muscles that need energy expenditure”^23^. Physical activity reduces blood pressure by decreasing sympathetic nerve activity and expanding artery lumen widths, thus lowering peripheral vascular resistance^24^. Moreover, physical activity can reduce the left ventricular mass index which can reduce blood pressure among people with hypertension^24,25^. Recent evidence from interventional research has found a strong link between regular physical activity and hypertension control^26–31^. However, some studies found no positive effect^32,33^. These contradictory findings could be due to interventional differences in the type of physical activity, intensity, adherence to physical activity, and study population. Similarly, meta-analyses that investigated the effect of physical activity in controlling hypertension have shown inconsistent results^34–37^. Meta-analyses were performed on studies that included either both normotensive and hypertensive people or limited to a specific age group^34^; limited to a certain form of physical activity such as walking, low-intensity aerobic exercise^35,36^; or overall exercise^37^.

Leisure-time physical activity (LTPA) is defined as “all behaviour related to physical activity that people take in their free time”^38^. The effects of LTPA in reducing cardiometabolic risk are higher than that of work-related physical activity^39^. LTPA such as walking, running, bicycling, and soccer are the top most popular physical activities among adults^40^, and many people can fit them into their daily routine. Findings from randomised controlled trials show that specific types of LTPA, such as walking^41^, soccer^42^, and swimming^43^, can reduce blood pressure. LTPA may be well positioned to promote cardiovascular health, particularly for those with hypertension, as they are popular behaviours that can be easily incorporated into public health messages. However, synthesised findings of the effect of all types of LTPA on blood pressure in only hypertensive people are lacking. Our objective in this study is to investigate the effect of LTPA on blood pressure control in people with hypertension.

## Methods

We followed the Preferred Reporting Items for Systematic Review and Meta-Analysis (PRISMA) to perform this systematic review^44^. This study is registered on PROSPERO (CRD42021260751). The protocol of this study has been published in a peer-reviewed journal^45^.

### Search methods

Our expert (KMSUR) on search strategy carried out comprehensive searches in Medline/PubMed, Cochrane Central Register of Controlled Trials (CENTRAL), Embase, Scopus, Web of Science, and Physical Education Index (ProQuest) on September 8, 2021, using the keywords and subject heading terms linked to study inclusion criteria (Suppl Appendix p. 1). We updated the search in all databases on February 14, 2023. We present the search method for databases in Suppl Appendix p. 1. We performed additional searches to identify more relevant articles by checking the reference list of included studies and reviews or systematic review articles we found during searching in databases. If required data was not supplied or full articles were not available, we contacted the corresponding author.

### Study selection criteria

We included both interventional and observational studies published since January 1, 2000. We followed the PICOS approach to select studies. We selected studies with only hypertensive people aged 18 and over. Any interventions on LTPA were included. The comparison was non-exercise performing controls (usual activities). The primary outcomes were SBP and DBP as continuous data. The secondary outcome was heart rate (beat per min). Interventions conducted to prevent hypertension were excluded. Details of the study selection criteria are in Appendix p. 6 and our published protocol^45^.

### Study selection

**Figure 1:**
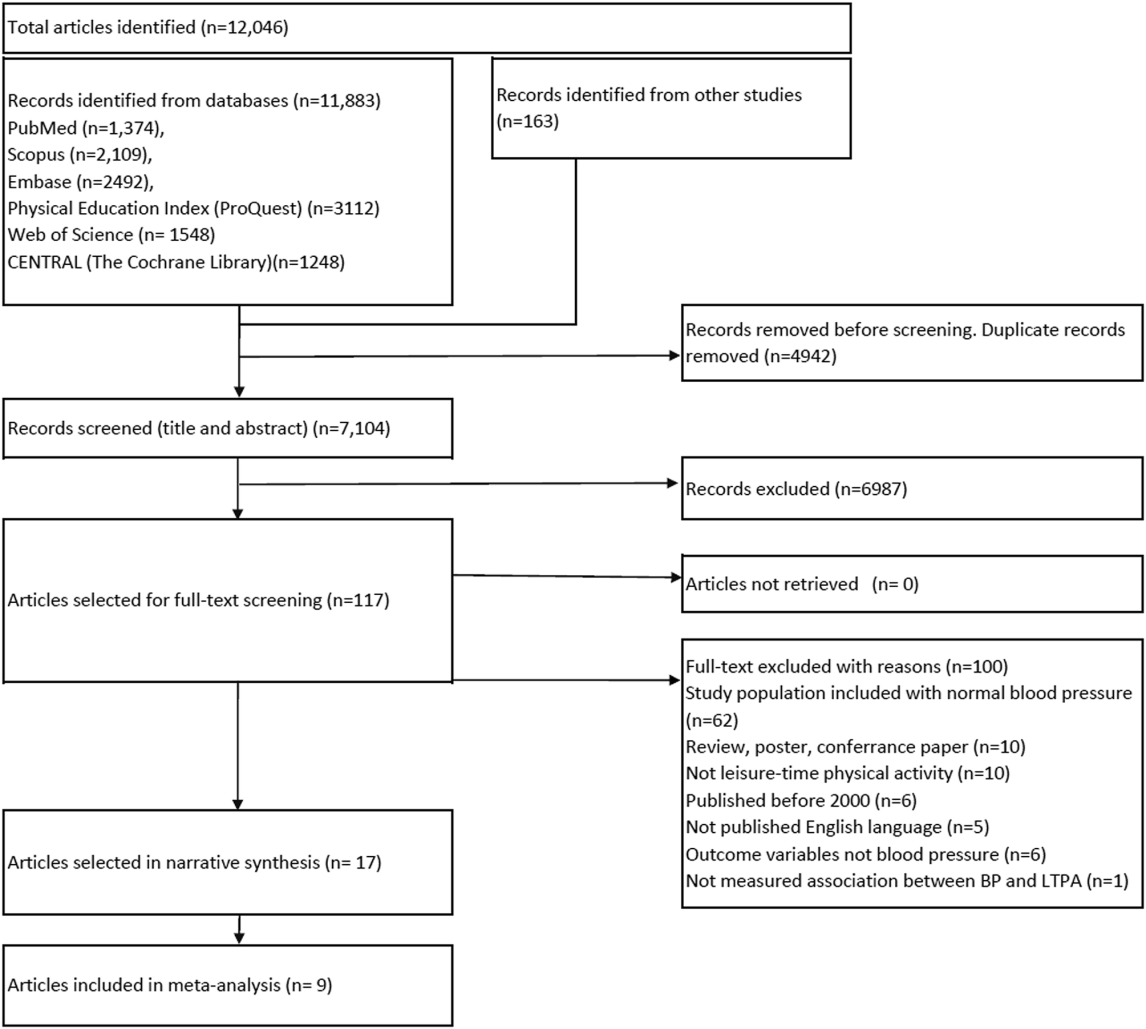
Process of systematic review (adopted from PRISMA 2020 statement^48^).

All identified articles were imported into Rayyan (an open-source software)^46^. Duplicate articles were removed. Two reviewers (AF and MGR) screened all the titles and abstracts independently, and discrepancies were resolved by discussion with a third reviewer (KMSUR). For the final selection of articles, an additional set of team members (MSI and MIS) independently examined the full text. Non-English papers were removed at the full-text assessment stage. The interrater reliability was 94.9% (kappa statistics 0.79) in full-text assessment. We used the prioritisation and sequential exclusion method during the screening of full-text articles^47^. We have illustrated the study selection process in the PRISMA flow chart (Fig. 1)^48^.

### Data extraction

Data extraction forms were developed based on the study population, methods, interventions, and findings on outcome variables. Two separate forms were used for observational and interventional studies. Two reviewers independently retrieved data from the included articles using the forms. Reviewers critically checked data consistency after completing data extraction, and disagreements were resolved through discussion.

### Assessment of risk of bias

Using the Joanna Briggs Institute (JBI) critical appraisal tools, two team members (RI and MSI) evaluated the methodological quality of the selected articles independently^49^. The interrater reliability was 90.9% (kappa statistics 0.75) in risk of bias assessments. The JBI tools for cross-sectional studies, cohort studies, and RCTs were used as appropriate. Any disagreements in this assessment were resolved by a third member (KMSUR).

### Data synthesis

#### Primary analysis

We followed American guidelines (minimum SBP 130 and DBP 80 mm Hg) of hypertension to define a participant as a hypertensive respondent. For our primary comparison, we performed a meta-analysis of all the interventional studies (quasi-experimental studies, randomised controlled trials, crossover trials) that had any type of LTPA interventions with moderate intensity, around 150 min of physical activity weekly, and a non-intervention control group. Moderate-intensity LTPA was considered to perform a meta-analysis as we got sufficient data on that in the included studies. We performed a meta-analysis on the effect of LTPA on SBP, DBP and heart rates. A narrative synthesis was performed for the rest of the included studies. We performed a random-effects model meta-analysis considering the heterogeneity. We also performed subgroup analysis for leisure-time walking intervention studies. The mean difference with the standard error was used in the meta-analysis. We produced a funnel plot and Egger's test to examine publication bias. We used the metaphor package to perform the meta-analysis in R-studio.

#### Sensitivity analysis

We conducted sensitivity analyses for all outcomes (SBP, DBP, and HR) by removing trials that had unclear random sequence generation and allocation concealment to examine the possible impact of risk of bias on the primary pooled estimates. We also undertook sensitivity analyses by including the crossover trials in the meta-analyses.

#### Certainty assessment

We followed the Grading of Recommendations, Assessment, Development, and Evaluation (GRADE) system to evaluate the evidence certainty for the intervention effect^50^. This integrates data from five main domains: risk of bias evaluation, heterogeneity, indirectness of evidence, the imprecision of findings (95% CI or small size of effect), and potential publication bias. Two reviewers graded the evidence independently as 'high quality', 'moderate', 'low', and 'very low quality'. We lowered the evidence from 'high certainty' by one level for serious constraints or by two levels for very serious study constraints. The overall certainty of the evidence is shown in Table 1.

**Table 1:** Summary finding of moderate-intensity leisure-time physical activity (all types) versus non-interventional control.

Types of leisure-time physical activity	Outcomes	Anticipated absolute effects (95% CI)	No of the participants (trials)	Certainty of the evidence (GRADE)^50^	Comments
		Effect with LTPA			
All types of moderate-intensity LTPA	Systolic blood pressure	MD −5.35 mm Hg ( −8.06 to −2.65)	531 (9 RCTs)	⊕ ⊕ ⊝ ⊝ Low^a^	Moderate-intensity LTPA may reduce systolic blood pressure
All types of moderate-intensity LTPA	Diastolic blood pressure	MD −4.76 mm Hg (−8.35 to −1.17)	531 (9 RCTs)	⊕ ⊕ ⊝ ⊝ Low^a^	Moderate-intensity LTPA may reduce diastolic blood pressure
Walking	Systolic blood pressure	MD −8.36 mm Hg (−13.39 to −3.32)	128 (3 RCTs)	⊕ ⊕ ⊝ ⊝ Low^b^	Walking may reduce systolic blood pressure
Walking	Diastolic blood pressure	MD −5.03 mm Hg (−8.23 to −1.84)	128 (3 RCTs)	⊕ ⊕ ⊝ ⊝ Low^b^	Walking may reduce diastolic blood pressure

*CI* confidence interval, *MD* mean difference, *LTPA* leisure-time physical activity, *RCT* randomized control trial.

^a^We downgraded one for inconsistency based on statistically significant heterogeneity. We downgraded one level for imprecision based on the size of individual studies and the range of confidence intervals. We did not downgrade for indirectness interventions of interest are directly comparable with the population of interest. Publication bias was undetected (Suppl Appendix p. 15, p. 36, and p. 37).

^b^Downgraded one-level inconsistency based on statistically significant heterogeneity. Downgraded one level for imprecision based on the size of individual studies and range of confidence interval. Not downgraded for indirectness and publication bias as interventions of interest are directly comparable with the population of interest. Publication bias was undetected. (Suppl Appendix p. 36, and p. 37).

### Role of the funding source

There was no funding for this study.

## Results

### Results of searches

We identified 12,046 articles in our searches. After methodically screening all the articles, 17 articles were included in this review ^41–43,51–64^ (Fig. 1). Out of the 17 selected articles, 12 were randomised control trials, two were quasi-experimental^42,55^, and three were cross-sectional studies^51,52,64^. Details and characteristics of studies are described in Suppl Appendix p.19.

### Characteristics of studies and participants

The included studies were conducted in 14 countries. Two studies were from the USA^59,62^, the UK^41,43^, and Brazil ^42,61^ each. Bangladesh^64^, China^51^, Hong Kong^60^, Indonesia^55^, Japan^57^, Korea^53^, Kuwait^52^, Iran^56^, Nepal^58^, New Zealand^63^, and Sweden^54^ had one study each. The age range of participants was 18 to 80. Out of 17 articles, 11 studies recruited both women and men respondents, while all participants were women in five studies^43,53,56,57,59^ and men in one study^42^.

### Intervention details

The types of LTPA in the interventions were walking (five studies)^41,42,59,62,63^, yoga (two studies)^54,58^, and one studies each on progressive muscle relaxation exercise^55^, stair climbing^53^, circuit training and chair-based exercise^57^, Quinton treadmill^56^, beach tennis^61^, swimming^43^, and qigong^60^. We classified walking, yoga, muscle relaxation exercise, stair climbing, moderate-intensity swimming, stair climbing as moderate-intensity exercise, and running, soccer, beach tennis, and high-intensity swimming as high-intensity exercise. The duration of exercise interventions ranged from six days to 26 weeks.

### Risk of bias in included studies

Details of the risk of bias have shown graphically in Suppl Appendix p. 16–18.

The total quality scores across all randomised controlled trials (RCT) ranged from 7 to 12 out of 13. The scores of the two selected quasi-experimental studies^42,55^ were 7 and 5 out of 9. The quality score in the three selected cross-sectional studies was 7 out of 8. The domains of quality assessed were inclusion criteria in the sample, details of study subjects and setting, reliability of exposure measurement, criteria of measurement, identification of confounders, strategies to deal with confounders, way of outcomes measurement, and appropriate statistical analyses. We assessed the reliability of exposure measurement as unclear in both studies. All other criteria were rated as yes in both studies.

### Other potential sources of bias

The funnel plots with Egger’s test in both SBP and DBP showed no publication bias (p < 0.05) (Suppl. Appendix p. 15).

## Effects of leisure-time physical activity

### Primary outcomes

#### Finding from meta-analysis

Out of the 12 RCTs, we performed a meta-analysis of studies on moderate intensity had a duration of 150 min of physical activity weekly, were not crossover trials and had a non-exercise control group. Nine studies met the inclusion criteria^41,43,53,54,56–59,62^.

**Figure 2:**
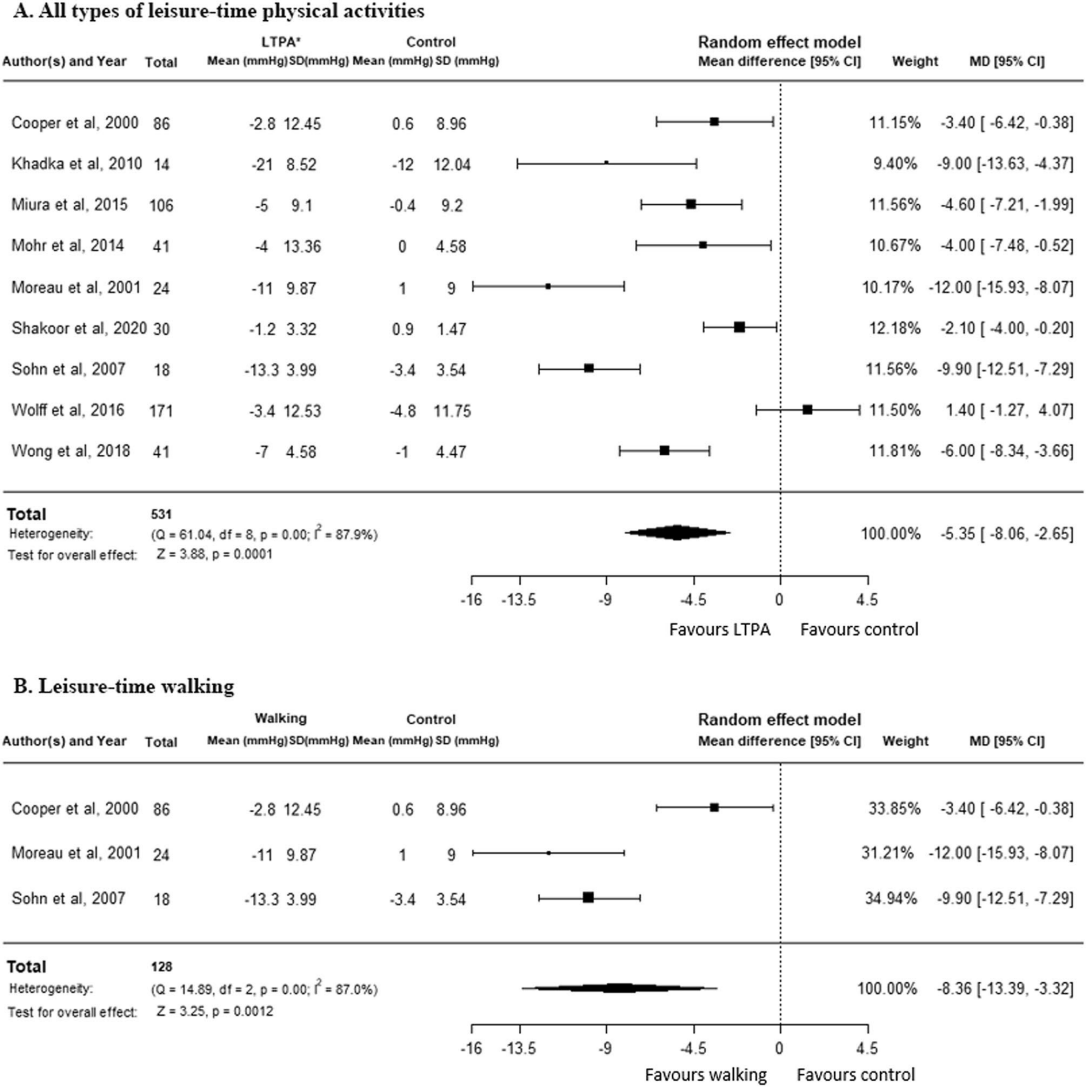
Moderate intensity leisure-time physical activities vs non-intervention control, outcome variable: systolic blood pressure. (**A**) All types of moderate intensity leisure-time physical activities vs non-intervention control . (**B**) Leisure-time walking vs non-intervention control.

Moderate-intensity LTPA (all types) reduced systolic blood pressure (SBP) compared to the non-intervention control group [mean difference (MD) −5.35 mm Hg, 95% confidence interval (CI) −8.06 to −2.65, P = 0.0001, I^2^ = 87.9%, nine trials, and n = 531] (Fig. 2A). Sensitivity analysis revealed a little larger effect when we pooled cross-over trials (MD −5.66 mm Hg, 95% CI −7.95 to −3.36, P < 0.0001, I^2^ = 84.9%, 11 trials, and n = 617) (Suppl Appendix p. 28), while the finding from parallel trials was statistically not significant (Suppl Appendix p. 33). The effect was reduced when we excluded studies with unsure randomization and allocation (MD −4.00 mmHg, 95% CI −7.70 to −0.30, P < 0.034, I^2^ = 91.2%, 11 trials, and n = 617) (Suppl Appendix p. 31). Subgroup analysis by only leisure-time walking showed a mean SBP reduction of −8.36 mmHg (95% CI −13.39 to −3.32, P = 0.0012, I^2^ = 87.0%, three trials, and n = 128) (Fig. 2B).

**Figure 3:**
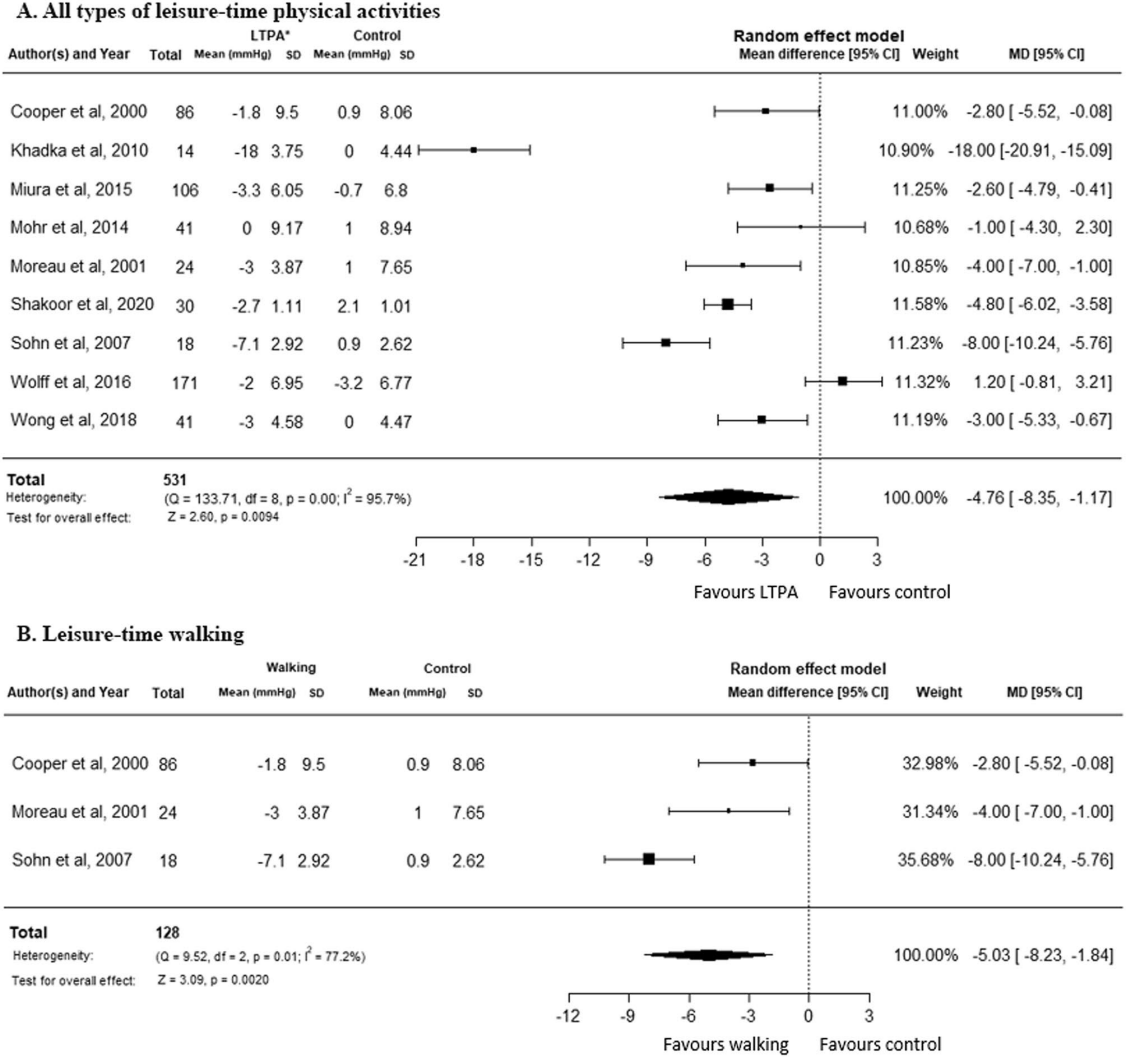
Moderate intensity leisure-time physical activities vs non-intervention control, outcome variable: diastolic blood pressure. (**A**) All types of moderate intensity leisure-time physical activities vs non-intervention control. (**B**) Leisure-time walking vs non-intervention control.

Mean diastolic blood pressure (DBP) reduced by −4.76 mm Hg, (95% CI −8.35 to −1.17, P = 0.0094, I^2^ = 95.7%, nine trials, and n = 531) in all types of moderate-intensity LTPA group compared to the non-interventions control group, nine studies, n = 531 (Fig. 3A). Sensitivity analysis revealed little difference when we included cross-over trials (MD −4.52 mmHg, 95% CI −7.52 to −1.58, p = 0.002, I^2^ = 94.2%, 11 trials and n = 617) (Suppl Appendix p. 29) and excluded studies with unsure randomization and allocation (MD −3.49 mmHg, 95% CI −6.43 to −0.54, p = 0.0202, I^2^ = 90.8%, five trials, and n = 346) (Suppl Appendix p. 32), while the finding from parallel trials was statistically not significant (Suppl Appendix p. 34). Walking during leisure time reduced mean DBP by −5.03 mm Hg, 95% CI −8.23 to −1.84, P = 0.02, I^2^ = 77.2%, three trials, n = 128) (Fig. 3B).

Overall, the evidence synthesised from the selected shows that moderate-intensity LTPA can reduce both systolic and diastolic blood pressure among adult people living with hypertension, however, the evidence is of low certainty as the GRADE approach (Table 1).

### Narrative synthesis

All interventional studies that were not included in the meta-analysis found that LTPA reduced both SBP and DBP significantly^42,60,61,63^. Cheung et al. (2005) compared the effect of conventional exercise with the effect of qigong in controlling hypertension and found a nonsignificant difference in SBP and DBP^60^. Nóbrega et al. (2013) compared the effect of walking with the effect of soccer in controlling hypertension and found a nonsignificant difference^42^. Three included cross-sectional studies also found that people who had a sedentary lifestyle had more likely higher blood pressure. Yang et al. (2019) found that 2 to 4 h of leisure-time daily walking was associated with lower DBP; however, the association was nonsignificant for SBP^51^. Alsairafi et al. (2010) found people who had to relax/do simple activities had more odds of uncontrolled hypertension than people who had moderate/vigorous activities^52^. Islam et al. (2023) found people who were involved in recreational sports had −3.9 mmHg lower SBP, however, the finding was not statistically significant^64^.

### Secondary outcomes

Moderate intensity LTPA (all types,) reduced mean heart rate by −2.69 beats/min (95% CI −4.80 to −0.58, p = 0.012, I^2^ = 66.9%, six trials, n = 256) (Suppl Appendix p. 35). The effect differed little in the sensitivity analysis. When we included cross-over trials, mean heart rate reduction was −2.94 (95% CI −4.76 to −1.13, p = 0.0015, I^2^ = 62.9%, seven trials and n = 318). The reduction of health rate was −1.94 (95% CI −3.38 to −0.49, p = 0.0085, I^2^ = 0%, two trials and n = 71) when we excluded unsure randomization and allocation studies (Suppl Appendix p. 33).

### Additional findings

Three studies examined the effect of LTPA in controlling mean atrial pressure^43,55,56^. Two studies reported that mean atrial pressure was reduced significantly^55,56^, while a study found that leisure-time swimming had an insignificant effect in controlling mean atrial pressure^43^.

## Discussion

The results of this meta-analysis of nine randomised controlled trials (531 people with hypertension) showed that moderate-intensity leisure-time physical activity significantly reduced blood pressure (both systolic and diastolic blood pressure) compared with non-intervention control (usual activities). The effect of LTPA exists even after undertaking sensitivity analyses excluding studies with unclear randomisation and allocation bias. LTPA also reduced heart rate. Furthermore, we found that leisure-time walking also has blood pressure and heart rate-lowering effects. Overall high-intensity physical activity (high-intensity group^56^ and soccer group^42^) reduced higher SBP than lower/moderate intensity physical activity (lower intensity group^56^ and walking group^42^). We found the amount of blood pressure (in mmHg) reduction was not dependent on the length of intervention. A short-term (10 days) intervention, brisk walking reduced −11.2 mmHg SBP^63^, while a long-term (26 weeks) intervention, walking reduced −8.5 mmHg SBP^62^.

Physical activity has been shown to improve a variety of biological processes related to reducing cardiovascular risk. Physical activity lowers blood pressure by reducing sympathetic nerve activity among people with hypertension^65^. It reduces the release of norepinephrine mediating vasoconstriction^66^, which in turn reduces vascular resistance. Physical activity increases insulin sensitivity and can reduce insulin-medicated sympathetic activity^65^. Physical activity reduces vascular responsiveness to endothelin-1, another vasoconstrictor among people with hypertension^67^. Physical activity also increases endothelial-dependent medicated vasodilation by enhancing nitric oxide production, and acetylcholine infusion. Physical activity causes vascular remodelling^68,69^. Vascular remodelling includes new arteries, increase cross-sectional area and enhancement of the diameter of existing veins and arteries. All of this vascular re-structure reduce peripheral resistance^70^. LTPA reduces heart rate. Bahrainy et al. reported the mechanism of physical activity and exercise to reduce heart rate is unclear^71^. They found an increase in parasympathetic tone or a reduction in beta-adrenergic stimulation due to physical activity does not reduce heart rate^72^. Tyagi et al. reported that an increased parasympathetic output can reduce heart rate after yoga^73^.

Our findings show that leisure-time physical activity reduced both SBP and DBP. The GRADE assessment shows the evidence has low certainty due to high heterogeneity among the RCTs and imprecision. The possible reason for heterogeneity is the type of LTPA, geographic location and sex of the participants. Subgroup analyses on geographic location and sex showed no to minimum heterogeneity (data not shown). The latitude, ambient temperature and solar radiation vary in geographic location. The effect of those factors on blood pressure can produce a heterogeneous response to LTPA^74^. Gender differences in LTPA on blood pressure can be due to differences in the metaboreflex, in insular gyral response, and in hormones (e.g. estrogen)^75,76^. The cause of the heterogenous effect of LTPA types on blood pressure is needed to explore. The included articles for meta-analysis did not classify the effect of LTPA on blood pressure based on the common effect modifiers for hypertension and LTPA, such as age, sex, body mass index, and socio-economic status. However, included articles considered those factors as confounders during analysis^41,54,57–59,62^.

A previous study reported that lowering blood pressure is associated with decreased mortality from cardiovascular diseases. A reduction of 2 mm Hg in SBP is associated with a 10% reduction in stroke mortality and a 7% reduction in vascular risk mortality^13^. We reported that moderate-intensity LTPA could reduce SBP MD by −5.35 mm Hg (95% CI: −8.06 to −2.65), which is a considerably greater magnitude than the 2 mm Hg reduction^13^. The findings thus can be considered clinically significant. It indicates that LTPA could serve as an effective and beneficial complement to or alternative to pharmacological treatment for lowering blood pressure in people with hypertension. The effect of physical activity does not sustain for the long term^77^, indicating the importance of regular physical activity. It is recommended that 150 min of physical activity a week^78^. We performed this meta-analysis of the intervention which examined physical activity length of 150 min per week. The finding of this study can be used to make LTPA recommendations for people with hypertension.

To the best of our knowledge, this is the first study that examines the effect of LTPA in lowering blood pressure among people with hypertension. However, a large number of systematic reviews and meta-analyses have investigated the effect of physical activity in reducing blood pressure^34,35,79–95^. Our review, like other systematic reviews, identifies a diverse group of physical activity interventions that differ in terms of participant willingness and intensity. For instance, aerobic exercise reduced both SBP (− 5.4 mm Hg) and DBP (− 3 mm Hg) among people with hypertension^37^. Another meta-analysis found moderate-intensity continuous training reduced SBP by 3.7 mm Hg and DBP by 2.41 mm Hg among people with hypertension^91^. Agreeing with another meta-analysis, this study suggests that LTPA reduces both SBP and DBP. We found walking also reduces SBP and DBP. The finding is coherent with the result of a Cochrane review^36^.

### Policy and practice implications

The findings of this study could be used to improve strategies and guidelines to recommend LTPA to reduce hypertension. The number of people with hypertension increased due to a recent change of practice guidelines designed by the American Heart Association^96,97^. Our findings from up-to-date evidence support the findings of the previous studies to offer LTPA as a treatment option, aside from the fact that the studies included in this meta-analysis did not directly evaluate the effects of exercise vs medicines^87,98,99^. As the findings are based on recreational activities among people living with hypertension, this nonpharmacological treatment option could increase adherence to continuing treatment among people with hypertension. This finding can be used to drive evidence-based conversations between patients and their clinicians regarding the benefits of exercise in decreasing blood pressure. This nonpharmacological intervention will reduce the treatment cost of hypertension, especially in countries that have limited resources and a shortage of medical services.

### Research implications

Studies conducted on a large number of people with hypertension are rare. Addressing this gap, the findings of this review will provide ethical justification to investigate the effectiveness of LTPA in reducing blood pressure among people living with hypertension. There is also a scope for future research to explore the barriers and facilitators of adherence to leisure-time physical activity among people with hypertension. In our included studies, Cheung et al. (2005)^60^ compared the effect of conventional exercise with the effect of qigong, and Nóbrega et al. (2013)^42^ compared the effect of walking with the effect of soccer in controlling hypertension. Both found LTPA reduced SBP and DBP; however, the mean difference was nonsignificant among the two comparison groups. It indicates the magnitude of the effectiveness of LTPA in controlling blood pressure varies little among the different types of LIPA. However, more evidence is needed to examine the effect difference by comparing different types of LTPA further.

### Strength and limitation

This systematic review and meta-analysis have several strengths. First, we have followed the standard robust method of conducting a systematic review and meta-analysis. We have performed meta-analysis only on randomised controlled trials, the gold standard for answering research questions.

This systematic review and meta-analysis has several limitations. The first limitation is that a good number of studies did not mention about randomisation process. We assessed the impact of unclear randomisation by performing a sensitivity analysis excluding studies that had unclear randomisation. We found a significant reduction of both SBP and DBP in the sensitivity analysis. Another limitation of this systematic review is that our search terms regarding leisure time (terms used in #1) were narrow. However, we have added a comprehensive list of LTPA in search term #2. It offsets the narrower strategy regarding the search term leisure time. The included studies in this meta-analysis had high heterogeneity. We performed a random effect model to address this potential limitation. We considered all types of LTPA together in meta-analysis. However, we have conducted several subgroup analyses and sensitivity analyses to overcome this. We included only articles published in the English language and removed five non-English articles during the full-text assessment. Finally, the small sample size of the majority included studies reduced the accuracy of the effect estimate.

## Conclusion

Moderate-intensity leisure-time physical activity may reduce both systolic and diastolic blood pressure (low certainty of the evidence) among people with hypertension. Moderate-intensity physical activity during recreational time can be promoted among people with high blood pressure to lower blood pressure. However, more studies are required to understand the effect difference among various types of LTPA in controlling blood pressure.

## Supplementary Information

Supplementary Information.

## Data Availability

We shared most of our data as an appendix. If further data is required, it is possible to share on request with the corresponding author, KM Saif-Ur-Rahman.
