# Supplementary material for: Effect of leisure-time physical activity on blood pressure in people with hypertension: a systematic review and meta-analysis
Source: Sci Rep. 2023 Jun 30;13:10639. doi: 10.1038/s41598-023-37149-2 (PMC10313796; doi:10.1038/s41598-023-37149-2)
Supplement: Supplementary file 1 — Supplementary Information. [file 41598_2023_37149_MOESM1_ESM.pdf]

Effect of Leisure-Time Physical Activity on Blood Pressure in Patients with Hypertension: A Systematic Review and Meta-Analysis

Authors: Md Shariful Islam[1], Ammatul Fardousi[2], Monaemul Islam Sizear[3,4], Md. Golam Rabbani[5], Rubana Islam[6], K.M. Saif-Ur-Rahman[2*]

Affiliation:

1. Infectious Diseases Division, icddr,b, Dhaka, Bangladesh
2. Health System and Population Studies Division, icddr,b, Dhaka, Bangladesh
3. Health System for Tuberculosis, Dhaka, Bangladesh
4. Public Health Foundation, Bangladesh, Dhaka, Bangladesh
5. Health Economics Unit, Health Services Division, Ministry of Health and Family Welfare, Dhaka, Bangladesh
6. Freelance researcher, Dhaka, Bangladesh

Corresponding Author: KM Saif-Ur-Rahman
Associate Scientist
Health Systems and Population Studies Division, icddr,b, Dhaka, Bangladesh
Email: su.rahman@icddrb.org

Appendix: Details of search strategies

We searched six databases on 8 September 2021.

We updated the search on 14 February 2023.

PubMed/Medline

Search Number	Search terms
**#1**	“Leisure time”[tiab] OR “playtime”[tiab] OR “recreation*”[tiab] OR “holiday”[tiab] OR “breaktime”[tiab] OR “free time”[tiab] OR “spare time”[tiab]
**#2**	Exercise[Mesh] OR exercise[tiab] OR “physical activ*”[tiab] OR sports[Mesh] OR sports[tiab] OR "Leisure Activities"[Mesh] OR “leisure activ*” [tiab] OR walking[Mesh] OR walking[tiab] OR running[Mesh] OR running[tiab] OR jogging[Mesh] OR jogging[tiab] OR baseball[Mesh] OR baseball[tiab] OR basketball[Mesh] OR basketball[tiab] OR bicycling[Mesh] OR bicycling[tiab] OR Boxing [Mesh] OR Boxing [tiab] OR "Cricket Sport"[Mesh] OR cricket[tiab] OR Dancing[Mesh] OR Dancing[tiab] OR football[Mesh] OR football[tiab] OR golf[Mesh] OR golf[tiab] OR Gardening[Mesh] OR Gardening[tiab] OR gymnastics[Mesh] OR gymnastics[tiab] OR mountaineering[Mesh] OR mountaineering[tiab] OR hockey[Mesh] OR hockey[tiab] OR "Martial Arts"[Mesh] OR "Martial Arts"[tiab] OR swimming[Mesh] OR swimming[tiab] OR volleyball[Mesh] OR volleyball[tiab] OR soccer[Mesh] OR soccer[tiab] OR “Water Sports”[Mesh] OR “Water Sports”[tiab] OR wrestling[Mesh] OR wrestling[tiab] OR “weight lifting”[Mesh] OR “weight lifting”[tiab] OR “track and field”[Mesh] OR “track and field”[tiab] OR skiing[Mesh] OR skiing[tiab] OR skating[Mesh] OR skating[tiab] OR “Snow Sports”[Mesh] OR “Snow Sports”[tiab] OR “Racquet Sports”[Mesh] OR “Racquet Sports”[tiab] OR tennis[Mesh] OR tennis[tiab] OR “physical fitness”[Mesh] OR “physical fitness” [tiab] OR “athletic performance”[Mesh] OR “athletic performance” [tiab] OR "Youth Sports"[Mesh] OR "Youth Sports"[tiab] OR “exercise training” [tiab]

**#3**	"Hypertension"[Majr] OR "Hypertension/rehabilitation"[Majr] OR "Hypertension/therapy"[Majr] OR Hypertension[tiab] OR "Blood Pressure" [Mesh] OR "Blood Pressure/classification"[Mesh] OR "Blood Pressure/complications"[Mesh] OR "Blood Pressure/diagnosis"[Mesh] OR "high blood pressure" [tiab] OR "Blood pressure" [tiab] OR blood pressure [tiab] OR "Blood Pressure/metabolism"[Mesh] OR "Blood Pressure/statistics and numerical data"[Mesh] OR "Blood Pressure/therapy"[Mesh] OR "high blood pressure" [tiab] OR "Blood pressure" [tiab] OR ((arterial OR diastolic OR systolic) AND pressure) OR "Blood Pressure" [tiab]
**#4**	#1 AND #2 AND #3

Embase

Search Number	Search terms
**#1**	'Leisure time'/exp OR 'playtime'/exp OR 'recreation'/exp OR 'holiday'/exp OR 'breaktime'/exp OR 'free time'/exp OR 'spare time'/exp
**#2**	'exercise'/exp OR 'physical activ*'/exp OR 'sports'/exp OR 'leisure activities'/exp OR 'walking'/exp OR 'running'/exp OR 'jogging'/exp OR 'baseball'/exp OR 'basketball'/exp OR 'bicycling'/exp OR 'boxing'/exp OR 'Cricket Sport'/exp OR 'dancing'/exp OR 'football'/exp OR 'golf'/exp OR 'gardening'/exp OR 'gymnastics'/exp OR 'mountaineering'/exp OR 'hockey'/exp OR 'Martial Arts'/exp OR 'swimming'/exp OR 'volleyball'/exp OR 'soccer'/exp OR 'water sports'/exp OR 'wrestling'/exp OR 'weight lifting'/exp OR 'track and field'/exp OR 'skiing'/exp OR 'skating'/exp OR 'Snow Sports'/exp OR 'Racquet Sports'/exp OR 'tennis'/exp OR 'physical fitness'/exp OR 'athletic performance'/exp OR 'Youth Sports'/exp

**#3**	'hypertension'/exp OR 'blood pressure'/exp OR 'high blood pressure'/exp OR 'bloodpressure'/exp OR 'arterial pressure'/exp OR 'diastolic pressure'/exp OR 'systolic pressure'/exp
**#4**	#1 AND #2 AND #3

Web of Science

Search Number	Search terms
**#1**	TS= ("Leisure time" OR "playtime" OR "recreation*" OR "holiday" OR "break time" OR "free time" OR "spare time")
**#2**	TS= (exercise OR "physical activ*" OR sports OR "leisure activ*" OR walk* OR running OR jogging OR baseball OR basketball OR bicycling OR Boxing OR cricket OR Dancing OR football OR golf OR Gardening OR gymnastics OR mountaineering OR hockey OR "Martial Arts" OR swimming OR volleyball OR soccer OR "Water Sports" OR wrestling OR "weight lifting" OR "track and field" OR skiing OR skating OR "Snow Sports" OR "Racquet Sports" OR tennis OR "physical fitness" OR "athletic performance" OR "Youth Sports")
**#3**	TS= (Hypertension OR "Blood Pressure" OR "high blood pressure" OR "arterial pressure" OR "diastolic pressure" OR "systolic pressure")
**#4**	#1 AND #2 AND #3

Scopus

Search Number	Search terms
**#1**	TITLE-ABS-KEY ("Leisure time" OR "playtime" OR "recreation*" OR "holiday" OR "break time" OR "free time" OR "spare time")

**#2**	TITLE-ABS-KEY (exercise OR “physical activ*” OR sports OR “leisure activ*” OR walk* OR running OR jogging OR baseball OR basketball OR bicycling OR Boxing OR cricket OR Dancing OR football OR golf OR Gardening OR gymnastics OR mountaineering OR hockey OR "Martial Arts" OR swimming OR volleyball OR soccer OR “Water Sports” OR wrestling OR “weight lifting” OR “track and field” OR skiing OR skating OR “Snow Sports” OR “Racquet Sports” OR tennis OR “physical fitness” OR “athletic performance” OR "Youth Sports")
**#3**	TITLE-ABS-KEY (Hypertension OR “Blood Pressure” OR “high blood pressure” OR “arterial pressure” OR “diastolic pressure” OR “systolic pressure”)
**#4**	#1 AND #2 AND #3

CENTRAL (The Cochrane Library)

Search Number	Search terms
**#1**	(“Leisure time” OR “playtime” OR “recreation*” OR “holiday” OR “break time” OR “free time” OR “spare time”)
**#2**	(exercise OR “physical activ*” OR sports OR “leisure activ*” OR walk* OR running OR jogging OR baseball OR basketball OR bicycling OR Boxing OR cricket OR Dancing OR football OR golf OR Gardening OR gymnastics OR mountaineering OR hockey OR "Martial Arts" OR swimming OR volleyball OR soccer OR “Water Sports” OR wrestling OR “weight lifting” OR “track and field” OR skiing OR skating OR “Snow Sports” OR “Racquet Sports” OR tennis OR “physical fitness” OR “athletic performance” OR "Youth Sports")

**#3**	(Hypertension OR “Blood Pressure” OR “high blood pressure” OR “arterial pressure” OR “diastolic pressure” OR “systolic pressure”)
**#4**	#1 AND #2 AND #3

Physical Education Index (ProQuest)

**Search Number**	**Search terms**
**#1**	(“Leisure time” OR “playtime” OR “recreation*” OR “holiday” OR “break time” OR “free time” OR “spare time”)
**#2**	(exercise OR “physical activ*” OR sports OR “leisure activ*” OR walk* OR running OR jogging OR baseball OR basketball OR bicycling OR Boxing OR cricket OR Dancing OR football OR golf OR Gardening OR gymnastics OR mountaineering OR hockey OR "Martial Arts" OR swimming OR volleyball OR soccer OR “Water Sports” OR wrestling OR “weight lifting” OR “track and field” OR skiing OR skating OR “Snow Sports” OR “Racquet Sports” OR tennis OR “physical fitness” OR “athletic performance” OR "Youth Sports")
**#3**	(Hypertension OR “Blood Pressure” OR “high blood pressure” OR “arterial pressure” OR “diastolic pressure” OR “systolic pressure”)
**#4**	#1 AND #2 AND #3

Appendix: Study selection criteria

Study settings

We selected studies that were conducted in community settings. We excluded studies that were performed in hospitals or healthcare facilities.

Type of studies

We included both interventional and observational studies published in the English Language. Any reviews, systematic reviews, reviews of reviews, or trial protocols were not included. Books, chapters, remarks, viewpoints, editorials, letters, correspondences, conference proceedings, and opinions were also excluded. We excluded studies published before 2000. We also excluded pre-prints. We included only studies published in the English language.

Type of participants

We selected studies with hypertensive participants aged 18 and over. We followed American guidelines (minimum SBP 130 and DBP 80 mm Hg) to define a participant as a hypertensive respondent. The participants with high blood pressure, whether controlled or uncontrolled, were included. Nationality, residency (urban or rural), sex, ethnicity, religion, area, and socioeconomic position of study participants were not used to exclude studies.

Type of interventions

Any physical activity interventions conducted in leisure time were included. Only findings reporting the effect of leisure-time physical activity were included if any research was undertaken to test the effect of overall physical activity on lowering blood pressure. The studies were discarded if the findings of leisure-time physical activity were not stated separately. Interventions conducted to prevent hypertension were excluded.

Comparators

The comparison was non-exercise performing controls.

Outcome measures

The primary outcomes were systolic blood pressure (SBP) and diastolic blood pressure (DBP) as continuous data. The blood pressure must be measured by any standard device (electronic or traditional mercury sphygmomanometer) in millimeters of mercury (mmHg). The secondary outcome was heart rate (beat per min).

Appendix: More details about the included articles

Funding Sources

The majority of studies (n=9) did not mention the funding sources of their studies. Six studies were funded by the government,[2,6,7,10-12] and one was funded by a charitable foundation.[5]

Adverse events

Out of the 14 interventional studies, only three trials reported information about adverse events.[5-7] Of the three reported studies, one study,[5] Cheung et al. (2005), reported an adverse event (A woman in the qigong group experienced vestibular neuritis).

Adherence

Adherence was measured in three out of the 14 included interventional studies. The reported adherence rates to the intervention were 100% in Shakoor et al. (2020),[13] 83.3% in Sulaeman et al. (2020),[8] and 78% in Wolff et al. (2016).[7]

Excluded studies

The main reasons to exclude studies were not including only hypertensive participants, not having blood pressure as an outcome, and not focusing on leisure-time physical activities. Details have been shared in appendix p. 8.

Appendix: Reasons for excluding articles

Articles	Reason for exclusion
Al-Hamdan et al. 2012	Not on hypertensive population only
Amigo 1997	Published before 2000
Aminian et al. 2021	Not on hypertensive population only
Andrews et al. 1982	Review article
Araiza 2006	Not on hypertensive population only
Archillaa et al. 2021	Not in the English language
Arija 2017	Not on hypertensive population only
Aro 1984	Not on hypertensive population only
Baker et al. 2008	Not on hypertensive population only
Baross et al. 2017	Normotensive population
Bayat et al. 2018	Normotensive population
Blom et al. 2011	Not on hypertensive population only
Blom et al. 2012	Not on leisure-time physical activity
Borhani 1996	Conference paper
Brandon et al. 2006	Not on hypertensive population only
Brown et al. 2014	Not on hypertensive population only
Bueno et al. 2016	The outcome variable was not controlling blood pressure
Byambasukh et al. 2018	Not on hypertensive population only
Byambasukh et al. 2019	Not on hypertensive population only
Byambasukh et al. 2020	Not on hypertensive population only
Casson et al. 2003	Not on leisure-time physical activity
Cederholm et al. 1986	Not on hypertensive population only

Articles	Reason for exclusion
Chaudhary et al. 1988	Published before 2000
Clays et al. 2012	Not on hypertensive population only
Crowley et al. 2021	Not measured association between Bp and LTPA
Delfino et al. 2020	Not on leisure-time physical activity
Dureja et al 2014	Not on hypertensive population only
Edimo et al. 2023	Not on hypertensive population only
Edla et al. 2016	Not on leisure-time physical activity
Elley et al. 2003	The outcome variable was not controlling blood pressure
Elmer et al. 1995	Not on leisure-time physical activity
Foulds et al. 2014	Normotensive population
Fritz et al. 2013	Not on hypertensive population only
Fuchs et al. 1993	Conference paper
Gallagher et al. 2021	Outcome variable not blood pressure
Gilson et al. 2007	Not on hypertensive population only
Ginsberg et al. 1990	Published before 2000
Gradidge et al. 2018	Not on hypertensive population only
Gupta 2019	Not on hypertensive population only
Haas et al. 2015	Not on hypertensive population only
Headley et al. 2017	Not on hypertensive population only
Hermart et al. 1994	Not on hypertensive population only
Herzig et al. 2014	Not on hypertensive population only
Isaacs et al. 2007	Not on hypertensive population only
Islam et al. 2021	Not on leisure-time physical activity
Islam et al. 2021 (2)	Not Leisure-time physical activity

Articles	Reason for exclusion
Joseph et al. 2019	The outcome variable was not controlling blood pressure
Kastarinen et al. 2007	Not on hypertensive population only
Klesges et al. 1991	Not on hypertensive population only
Kibria et al. 2022	Outcome variable not blood pressure
Koh et al. 2010	Not on hypertensive population only
Koh et al. 2010	Not on hypertensive population only
Kríz et al. 1971	Not in the English language
Kríz et al. 1971	Not in the English language
Kurban et al. 2011	Not on hypertensive population only
Lawton et al. 2008	Not on hypertensive population only
Lee et al. 2011	Not on hypertensive population only
Li et al. 2021	Not on hypertensive population only
LIU 2001	The outcome variable was not controlling blood pressure
Liu et al. 2022	Not on hypertensive population only
Lowensteyn et al. 2016	Poster presentation
Miles et al. 2013	Not on hypertensive population only
Millett et al. 2013	Not on leisure-time physical activity
Milon et al. 1986	Not on hypertensive population only
Mozos et al. 2015	Not on hypertensive population only
Murphy et al. 2006	Normotensive population
Nemoto et al. 2007	Normotensive population
Palatini 2012	Review article
Pandey et al. 2017	Poster presentation
Pecelj-Gec et al. 1990	Not in the English language; excluded

Articles	Reason for exclusion
Penco et al. 2006	Review article
Pescatello et al. 2004	Review article
Petal 1975	Published before 2000
Petal 1975	Published before 2000
Petal 1975	Published before 2000
Pitanga et al. 2010	Not on hypertensive population only
Premila 2017	Not on leisure-time physical activity
Pressman et al. 2009	Not on hypertensive population only
Rasmussen et al. 2018	Not on hypertensive population only
Reaven et al. 1991	Not on hypertensive population only
Ribeiro Junior 2020	Not on hypertensive population only
Richter-Heinrich et al. 1980	Not in the English language
Riegel et al. 2019	Not on hypertensive population only
Riemenschneider et al. 2020	Not on hypertensive population only
Saensak et al. 2013	Not on leisure-time physical activity
Sakuragi et al. 2006	Normotensive population
Sarma 2015	Not on hypertensive population only
Sousa et al. 2013	Conference paper
Stoltzfus et al. 2020	Normotensive population
Su et al. 2021	Not on hypertensive population only
Takata et al. 2010	Not on hypertensive population only
Treff et al. 2017	Not on hypertensive population only
Tsao et al. 2022	Not on hypertensive population only
Tudor-Locke et al 2004	Not on hypertensive population only

Articles	Reason for exclusion
Vriz et al. 2022	Not on hypertensive population only
Wang et al. 2021	Not on hypertensive population only
Werneck et al. 2021	Not on hypertensive population only
Werneck et al. 2018	Not on hypertensive population only
Yao et al. 2017	Supplementary publication. Authors contacted
Zubir et al. 2022	Not on hypertensive population only

Appendix: Risk of bias assessment using JBI critical appraisal tools

Randomised controlled trials

Random sequence generation and allocation and concealment of treatment were used in 67% (n=8/12) trials, while they were unclear in the rest of the four RCTs.[1-4] Treatment groups were similar at the baseline in 92% (n=11/12) of the trials. Due to the nature of interventions, it was difficult to maintain blinding in both participants and personnel delivering treatment. We judged it as not applicable in those cases. Only two studies maintained blinding of treatment to participants.[5,6] We found that 92% (n=11/12) of trials did not share adequate information on blindness treatment assignment with outcome assessors. One study maintained blinding of treatment assignment to outcome assessors.[7] In all the trials, treatment groups were treated the same other than the intervention of interest. We assessed 'complete follow up' as yes in 5/12 studies, no in 4/12, and unclear in 3/12 studies. Participants were analysed in the groups to which they were randomised in 83% (n=10/12) of the trials, and for the two crossover trials, we judged it as not applicable. We measured 'outcomes in an identical way for treatment groups' as yes in 83% (n=10/12) and unclear in 17% (n=2/12) of the RCTs. 'Outcomes measured reliably' were judged as yes in 83% (n=10/12) and unclear in 17% (n=2/12) of RCTs. A total of 92% (n=11/12) of trials used appropriate statistical analysis, while we found one study did not provide adequate information about this. The trial design was appropriate for all the RCTs.

Other studies

'Cause and effect' were clear in both quasi-experimental studies. Both studies had similar comparison groups. However, participants included in the comparison groups receiving similar treatment were unclear in both studies. Sulaeman et al (2020)[8] had a control group, whereas Nóbrega et al (2013)[9] did not. Sulaeman et al. (2020)[8] did not perform multiple measurements of the outcome both pre-and post-intervention/exposure, while we assessed it for Nóbrega et al. (2013)[9] as not applicable. Follow-up was completed by Nóbrega et al. (2013)[9]; however, it was unclear by Sulaeman et al. (2020)[8]. Outcomes were measured in the same way in both studies, while outcomes measured reliably were unclear in Sulaeman et al. (2020)[8]. We judged that Nóbrega et al. (2013)[9] used appropriate statistical analysis, while Sulaeman et al. (2020)[8] did not.

A. Randomized control trial (n=12)

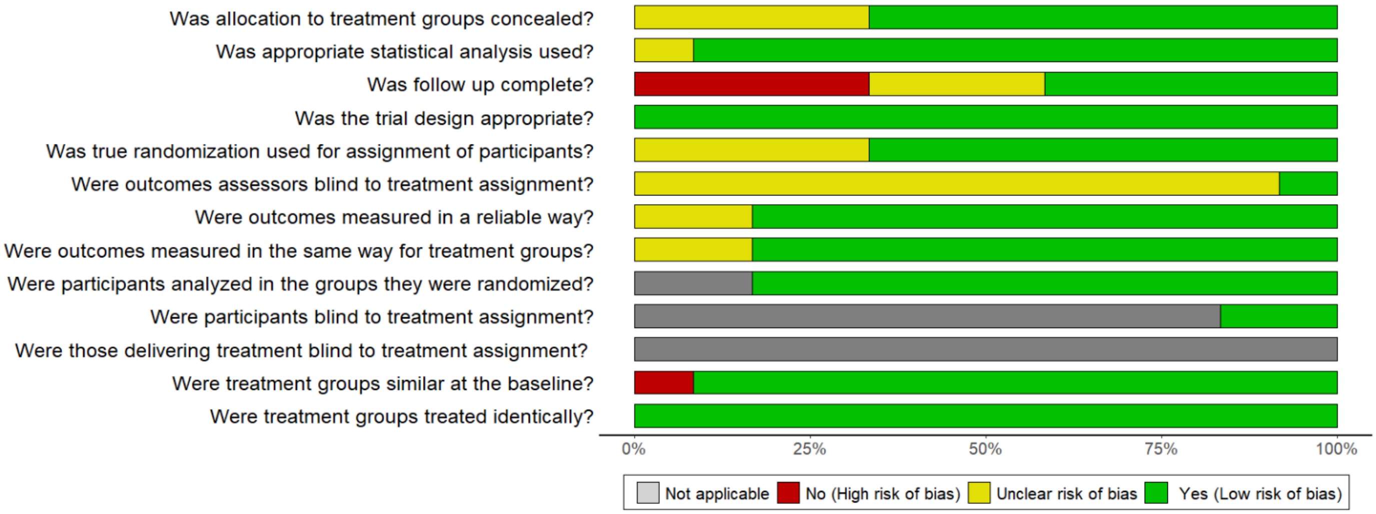

[illegible]

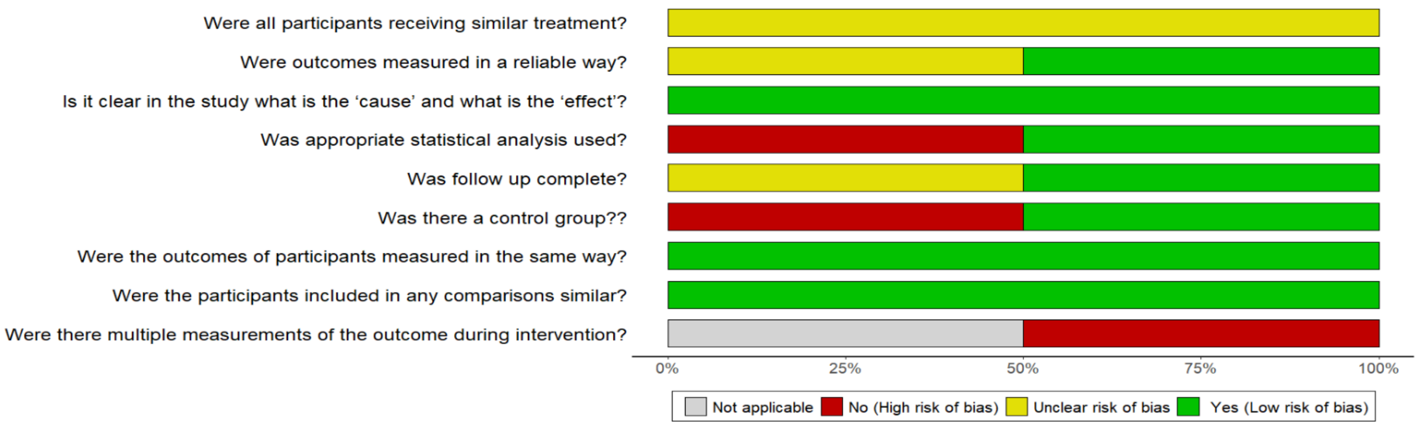

C. Cross-sectional study (n=3)

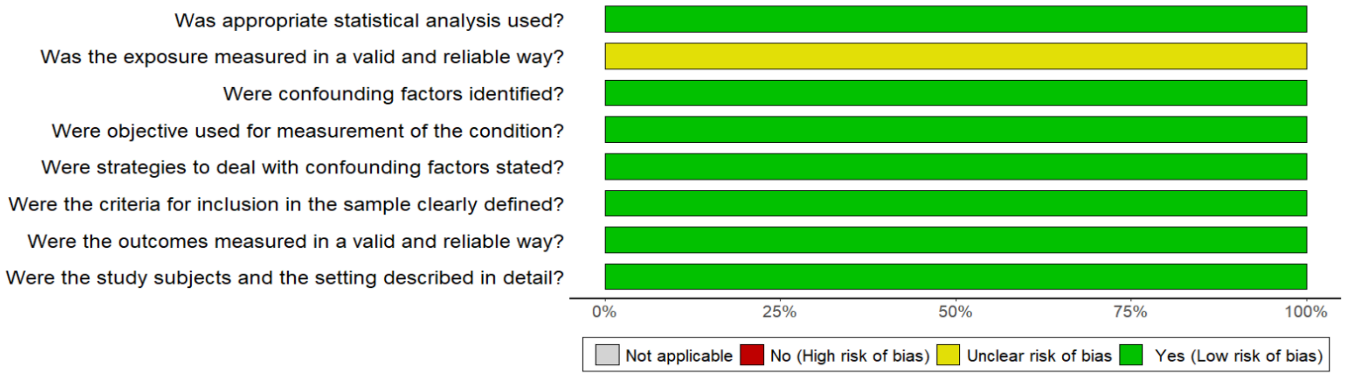

Appendix: Funnel plot with Egger's test for publication bias

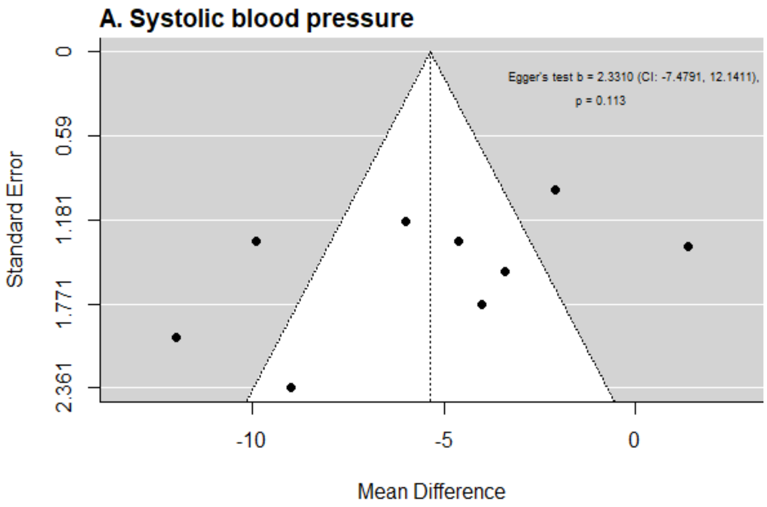

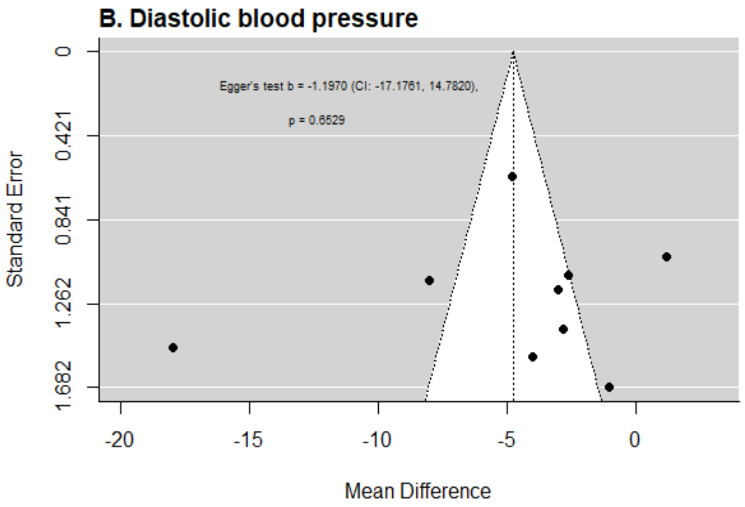

Appendix: Risk of bias score domain of randomized controlled trials measured using JBI critical appraisal tool

**Authors & year**	**1.Was true randomization used for assignment of participants to treatment groups?**	**2.Was allocation to treatment groups concealed?**	**3. Were treatment groups similar at the baseline?**	**4. Were participants blind to treatment assignment?**	**5. Were those delivering treatment blind to treatment assignment?**	**6. Were outcomes assessors blind to treatment assignment?**	**7. Were treatment groups treated identically other than the intervention of interest?**	**8. Was follow up complete and if nt, were differences between groups in terms of their follow up adequately described and analyzed?**	**9. Were participants analyzed in the groups to which they were randomized?**	**10. Were outcomes measured in the same way for treatment groups?**	**11. Were outcomes measured in a reliable way?**	**12. Was appropriate statistical analysis used?**	**13. Was the trial design appropriate, and any deviations from the standard RCT design (individual randomization, parallel groups) accounted for in the conduct and analysis of the trial?**	**Score (13)**
**Carpes et al (2021)**	Yes	Yes	Yes	Yes	NA	Unclear	Yes	Yes	NA	Yes	Yes	Yes	Yes	12
**Cheung et al (2005)**	Yes	Yes	Yes	Yes	NA	Unclear	Yes	Yes	Yes	Yes	Yes	Yes	Yes	12
**Cooper et al (2000)**	Yes	Yes	Yes	NA	NA	Unclear	Yes	No	Yes	Yes	Yes	Yes	Yes	11
**Scott et al (2006)**	Yes	Yes	Yes	NA	NA	Unclear	Yes	No	NA	Yes	Yes	Yes	Yes	11
**Khadka et al (2010)**	Unclear	Unclear	Yes	NA	NA	Unclear	Yes	Unclear	Yes	Unclear	Unclear	Unclear	Yes	7
**Miura et al (2015)**	Unclear	Unclear	Yes	NA	NA	Unclear	Yes	Unclear	Yes	Unclear	Unclear	Yes	Yes	7
**Mohr et al (2014)**	Unclear	Unclear	Yes	NA	NA	Unclear	Yes	Yes	Yes	Yes	Yes	Yes	Yes	10
**Moreau et al (2001)**	Unclear	Unclear	Yes	NA	NA	Unclear	Yes	Yes	Yes	Yes	Yes	Yes	Yes	10
**Sohn (2007)**	Yes	Yes	No	NA	NA	Unclear	Yes	Yes	Yes	Yes	Yes	Yes	Yes	12
**Sulaeman et al (2020)**	Yes	Yes	Yes	NA	NA	Unclear	Yes	Unclear	Yes	Yes	Yes	Yes	Yes	11
**Wolff et al (2016)**	Yes	Yes	Yes	NA	NA	Yes	Yes	No	Yes	Yes	Yes	Yes	Yes	12
**Wong et al (2018)**	Yes	Yes	Yes	NA	NA	Unclear	Yes	No	Yes	Yes	Yes	Yes	Yes	11

Appendix: Risk of bias score domain of quasi-experimental studies measured using JBI critical appraisal tool

Authors & year	1. Is it clear in the study what is the ‘ cause’ and what is the ‘ effect’ (i.e. there is no confusion about which variable comes first)?	2. Were the participants included in any comparisons similar?	3. Were the participants included in any comparisons receiving similar treatment/care, other than the exposure or intervention of interest?	4. Was there a control group?	5. Were there multiple measurements of the outcome both pre and post the intervention/exposure?	6. Was follow up complete and if not, were differences between groups in terms of their follow up adequately described and analyzed?	7. Were the outcomes of participants included in any comparisons measured in the same way?	8. Were outcomes measured in a reliable way?	9. Was appropriate statistical analysis used?	Score (9)
**Nóbrega et al (2013)**	Yes	Yes	Unclear	No	na	Yes	Yes	Yes	Yes	7
**Sulaeman et al (2020)**	Yes	Yes	Unclear	Yes	No	Unclear	Yes	Unclear	No	5

Appendix: Risk of bias score domain of cross-sectional studies measured using JBI critical appraisal tool

Authors & year	1. Were the criteria for inclusion in the sample clearly defined?	2. Were the study subjects and the setting described in detail?	3. Was the exposure measured in a valid and reliable way?	4. Were objective, standard criteria used for measurement of the condition?	5. Were confounding factors identified?	6. Were strategies to deal with confounding factors stated?	7. Were the outcomes measured in a valid and reliable way?	8. Was appropriate statistical analysis used?	Score (8)
**Alsairafi et al (2010)**	Yes	Yes	Unclear	Yes	Yes	Yes	Yes	Yes	7
**Islam et al (2023)**	Yes	Yes	Unclear	Yes	Yes	Yes	Yes	Yes	7
**Yang et al (2019)**	Yes	Yes	Unclear	Yes	Yes	Yes	Yes	Yes	7

Appendix: Characteristics of included articles (interventional studies) (1/2)

Study	Aim	Country	Study design	Sample size (randomized)	Number of completers	Sex of participants	Age of participants	Smoking status
**Carpes et al (2021)**	To evaluate the effect of a beach tennis session on 24-h ambulatory blood pressure in adults with hypertension.	Brazil	Randomized crossover trial	24	24	M- 12, F-12	35 to 60 years	No smokers
**Cheung et al (2005)**	To study the effect of Guolin Qigong on blood pressure. Qigong is a traditional Chinese exercise consisting of breathing and gentle movements.	Hong Kong	Randomized controlled trial	88 (Qigong 47, Conventional Exercise 41)	Qigong (37), Conventional Exercise (36)	M- 37, F- 51	18 to 75 years	5 smokers (3 in the intervention arm, 2 in the control arm)
**Cooper et al (2000)**	To investigate the effect of a six-week program of moderate-intensity exercise on daytime ambulatory blood pressure among unmedicated, sedentary adults aged 25 years to 63 years with the office blood pressure of 150 mmHg to 180 mmHg systolic and/or 91 mmHg to 110 mmHg diastolic.	UK	Randomized controlled trial	90 (IN-48, CON-42)	86 (In-47, CON-39)	M-72, F-18	25 to 63 years	Not mentioned
**Scott et al (2006)**	To assess whether four 10-minute ‘snacks’ of exercise per day are as effective at lowering blood pressure as 40 minutes of continuous moderate exercise, when compared with no exercise.	New Zealand	Single-blind randomised crossover trial	35	31	M- 16, F-19	Mean age 53	Not mentioned
**Khadka et al (2010)**	To investigate the effect of yoga on cardiovascular autonomic reactivity in essential hypertensive patients.	Nepal	Randomised controlled trial	14 (IN-7, CON-7)	14	M, F	30 to 60 years	Not mentioned
**Miura et al (2015)**	The aim of this study was to compare the effects of exercise training on arterial stiffness between older hypertensive and healthy females.	Japan	Randomised controlled trial	221	221	F	66 to 88 years	Not mentioned

Study	Aim	Country	Study design	Sample size (randomized)	Number of completers	Sex of participants	Age of participants	Smoking status
**Mohr et al (2014)**	The objective of the present study was to test the hypothesis that high-intensity swim training is an efficient strategy to reduce blood pressure and improve the cardiovascular health profile in sedentary premenopausal women with mild to moderate hypertension	UK	Randomized controlled trial	62 (HIT IN-21, MOD IN-21, CON-20)	62	F	35 to 49	Not mentioned
**Moreau et al (2001)**	The present study tested the hypothesis that walking activity following the ACSM-CDC physical activity recommendation would lower BP in postmenopausal women with high BP	USA	Randomized controlled trial	24 (IN- 15, CON-9)	24	F	51 to 56	non-smokers
**Nóbrega et al (2013)**	To compare the acute pressoric response of a soccer match with a walking/running session in recreational soccer practices.	Brazil	Quasi-experimental	8	8	M	40 to 60 years	Not mentioned
**Shakoor et al (2020)**	This study aimed to investigate the Acute HIIT and IHG exercise on hemodynamic responses (blood pressure [systolic, diastolic, mean arterial pressure], rate pressure product and heart rate) in women with hypertension.	Iran	Randomised controlled trial	45 (HIIT IN-15, IHG IN-15, CON-15)	45	F	45±5 years	Non-smoker
**Sohn(2007)**	To determine whether the encouragement of walking an extra 30 minutes a day decreases blood pressure in adult African Americans with newly diagnosed hypertension.	USA	Randomized controlled study.	19 (IN-9, CON-10)	18 (IN-8, CON-10)	M- 6, F- 12	25 to 59 years	Not mentioned
**Sulaeman et al (2020)**	To assess the effectiveness of family training towards the regularity of progressive muscle relaxation exercise implementation in hypertension patients.	Indonesia	quasi-experimental study	60	60	M-17, F-43	26 to 55	Not mentioned

Study	Aim	Country	Study design	Sample size (randomized)	Number of completers	Sex of participants	Age of participants	Smoking status
**Wolff et al (2016)**	The present study was designed to evaluate yoga's impact on blood pressure (BP) and quality of life (QOL) and on stress, depression and anxiety in patients with hypertension in a primary care setting.	Sweden	Multi-centre parallel group randomized controlled trial	191 (IN- 96, CON- 95)	171 (IN- 85, CON- 86)	M- 44, F- 52	30 to 80	Not mentioned
**Wong et al (2018)**	To examine the effects of a stair climbing (SC) regimen on arterial stiffness (pulse wave velocity [PWV]), BP, and leg strength in postmenopausal women with stage 2 hypertension	Korea	Parallel experimental design	41 (IN-21, CON-20)	41	F	49-67 years	No smokers

Appendix: Characteristics of included articles (interventional studies) (2/2)

Study	Intervention	Duration	Intensity	Frequency	Intervention period	Outcome variables	Adherence	Adverse event	Note	ROB score	MA
**Carpes et al (2021)**	Beach tennis	45 mins	Followed high, moderate & vigorous intensity	Total twice	5-10 days	SBP, DBP	Not mentioned	No adverse event happened	This study was partially funded by the Research and Education Fund of the Hospital de Clínicas de Porto Alegre (FIPE/ HCPA, Grant number 18-0642).	12 out of 13	No

**Cheung et al (2005)**	Goulin qigong	Qigong for 60 min in the morning and 15 min in the evening every day for the duration of the study, comprising walking exercises for 40 min, eight sets of stretching exercises and three sets of pressure-relieving exercises in the morning and three sets of pressure-relieving exercises in the evening.	Moderate	Twice everyday	16 weeks	SBP, DBP	Not mentioned	A woman in the qigong group experienced vestibular neuronitis that was not related to the practice of qigong.	The study was funded by a charitable donation for research from the Li Ka Shing Foundation. The Foundation is not involved in the design, conduct, analysis and reporting of the study.	12 out of 13	No
**Cooper et al (2000)**	Walking	30 minutes daily	Moderate	5 days per week	Six weeks	SBP, DBP	Not mentioned	Not mentioned	This study was funded by the NHS Executive South and West Research and Development Directorate.	11 out of 13	Yes

**Scott et al (2006)**	Walking	40 mins per day, 4*10 minutes walking	Moderate	once in a day	10 days	SBP, DBP	Not mentioned	Not mentioned	The initial study was funded by the Health Research Council of New Zealand. Phase 3 of the study was funded by Child Health Research Foundation, Becroft Foundation, and Auckland Medical Research Foundation. It was conducted in the Children's Research Centre, which the Starship Foundation support Auckland Healthcare Ltd	11 out of 13	No
**Khadka et al (2010)**	Different kinds of Yoga practices (Strengthening exercise, Yogic asanas, Shavasana, Meditation & Pranayama)	30 mins in a day. Strengthening exercise - 5mins Yogic asanas- 6/7 mins Shavasana - 5 mins Meditation- 5 mins Pranayama - 4 mins	Moderate	Six days in a week	Six weeks	SBP, DBP	Not mentioned	Not mentioned		7 out of 13	Yes

**Miura et al (2015)**	Traning: 1. resistance exercises for circuit training and 2. chair-based exercise for the lower extremities.	90 minutes daily	Moderate	Twice a week	12 weeks	SBP, DBP, HR	Not mentioned	Not mentioned	This study was supported by a grant-in-aid for Scientific Research from the Ministry of Education, Science, Sports and Culture of Japan (15700441).	7 out of 13	Yes
**Mohr et al (2014)**	Swimming	HIT: 15-25mins MOD: 1 hour daily	HIT: High-intensity swimming MOD: Moderate intensity swimming	three times per week	15 weeks	SBP, DBP, HR	Not mentioned	Not mentioned		10 out of 13	Yes
**Moreau et al (2001)**	Walking	3km/day or more than their lifestyle of walking	Moderate	Once a day	24 weeks	SBP, DBP, HR	Not mentioned	Not mentioned		10 out of 13	Yes
**Nóbrega et al (2013)**	Soccer & walking/running	Soccer - 60 mins & Walking/running - 60 mins	High	Once a day	one day	SBP, DBP	Not mentioned	Not mentioned		7 out of 13	No
**Shakoor et al (2020)**	High-intensity interval training (HIIT): Quinton treadmill; and isometric handgrip (IHG) exercise: Isometric contractions	HIIT: 4×4 min intervals at 85-95% peak HR, interspersed with a 3-min active recovery period at ≈70% Peak HR. IHG: 4×2 min by 2 minutes rest	Moderate	_	_	SBP, DBP	All the participants completed their training program	Not mentioned	This study was approved by the Research Ethics Committee of Shiraz University Of Medical Sciences-Iran (IR.SUMS.REHAB. REC.1398.001)	5 out of 13	Yes

**Sohn(2007)**	Walking	30 minutes daily	Moderate	once in a day	26 weeks	SBP, DBP	Not mentioned	Not mentioned	The study was partly funded by the Establishment of Departments of Family Medicine Grant No. 5 d32HP10234 from the Health Resources and Services Administration.	12 out of 13	Yes
**Sulaeman et al (2020)**	Progressive muscle relaxation exercise technique consisted of 16 movements	32-48 mins	Moderate	twice a day	6 days	Mean Arterial Pressure	90% doing the exercise regularly in the training meanwhile, two people doing the exercise regularly but did not follow the training	Not mentioned	The study was approved by the medical ethical commission of the University of Mataram and informed consent was taken from all participants	11 out of 13	No

**Wolff et al (2016)**	Home based Kundalini yoga	15 minutes	Moderate	Twice daily	12 weeks	SBP, DBP	Adherence to the yoga intervention was 78%	There were no serious adverse events reported by the yoga group participants.	This study was funded by the Faculty of Medicine at Lund University, the Ekhaga Foundation, the Swedish Heart-Lung Foundation and the Swedish Southern Health Care Region, Agreement for Medical Education and Research (ALF) funding from Region Skåne and a Swedish Research Council grant awarded to Kristina Sundquist.	12 out of 13	Yes
**Wong et al (2018)**	Stair climbing	192 steps	Moderate	Four days a week in weeks 1 and 2, By the last three weeks (10-12)- 4 days a week	12 weeks	baPWV, SBP, DBP	Not mentioned	Not mentioned	All protocols were approved by the Institutional Review Board of the Public Institutional Review Board designated by the Ministry of Health and Welfare (SH-IRB 2017-13), registered in Clinicaltrials.gov (NCT03254251)	11 out of 13	Yes

Appendix: Characteristics of included articles (observational studies)

Study	Aim	Country	Study design	Sample size	Sex of participants	Age of participants	Smoking?	Type of LTPA	Outcome variables	ROB score	MA
**Alsairafi et al (2010)**	To demonstrate the effect of physical activity on controlling blood pressure among hypertensive patients from the Mishref area of Kuwait.	Kuwait	Cross-sectional	240	M-87, F-148	Mean age 48.2+/- 9 SD	Smoking-37	Moderate/vigorous activity & relax/simple activity	SBP, DBP	7 out of 8	No
**Islam et al. (2023)**	Aim to investigate the associations of current physical activity levels, sedentary time, knowledge of and attitude towards physical activity with blood pressure in people with hypertension in a rural area in Bangladesh.	Bangladesh	Cross-sectional	307	M-153, F-154	30 to 75 years	Smoking-62	Moderate intensity	SBP, DBP	7 out of 8	No
**Yang et al (2019)**	The associations between various LTDW durations and BP readings were examined among Chinese older adults. The association between LTDW time and BP outcomes were examined in Chinese older adults with hypertension.	China	Cross-Sectional	780	M-383, F-397	>65 years	Nonsmoker-455<br>Former Smoker -109<br>Current smoker - 216	Daily walking	SBP, DBP	7 out of 8	No

Appendix: Sensitivity analysis 1: Leisure-time physical activity versus control, including crossover trials, Outcome- Systolic blood pressure*

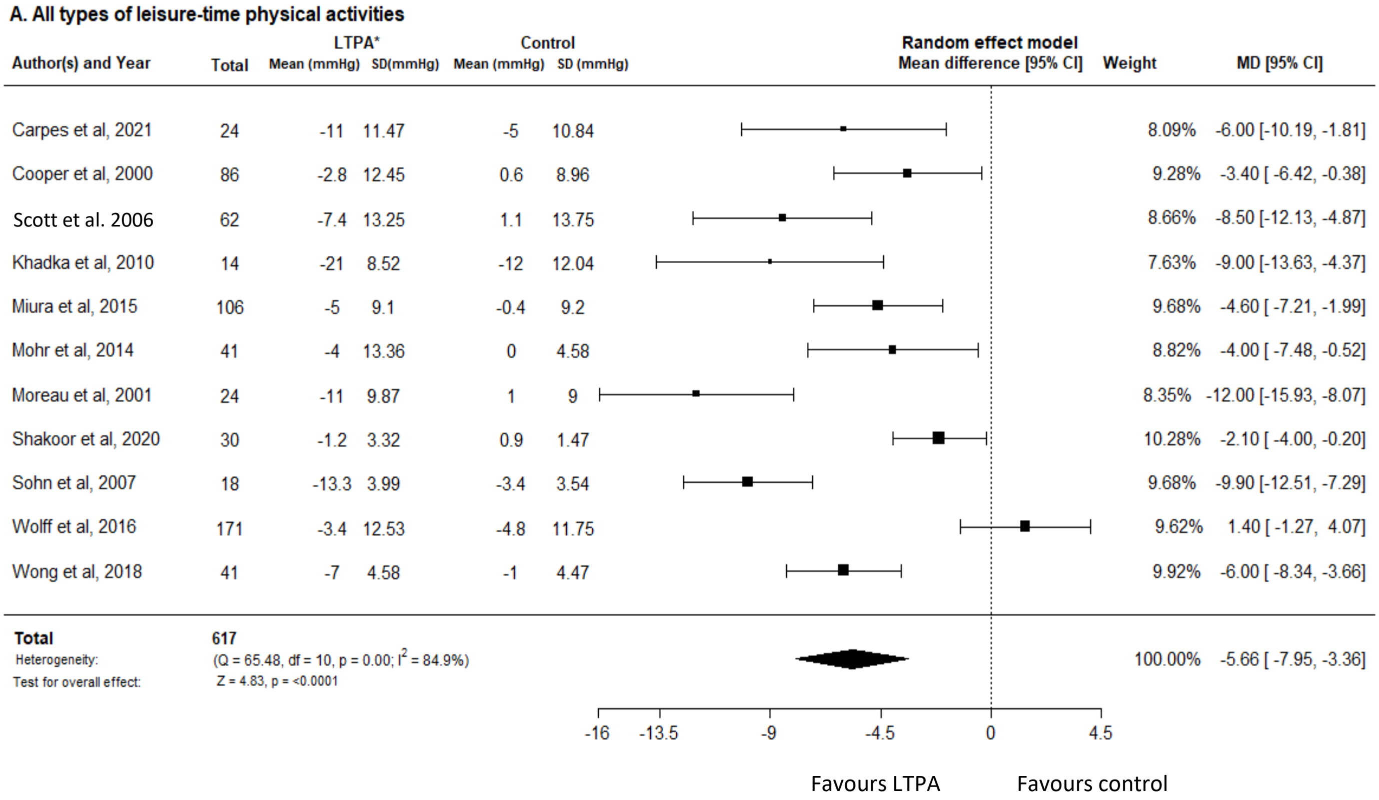

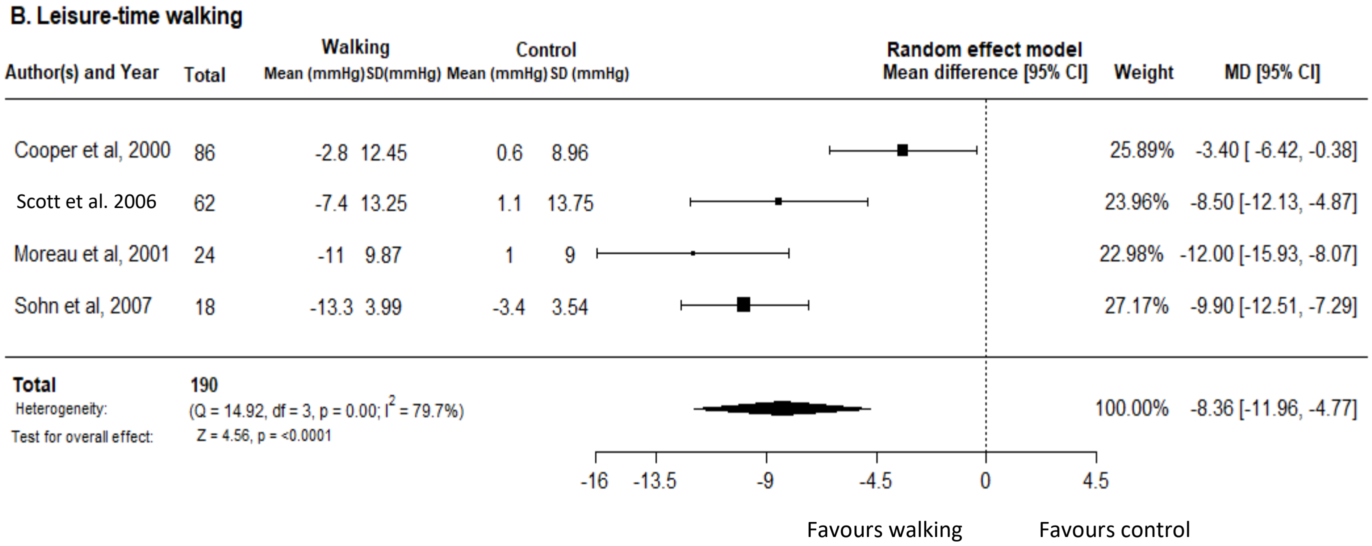

*Carpes et al. 2021 examine the effect of high-intensity exercise, beach tennis on lowering blood pressure.

Appendix: Sensitivity analysis 1: Leisure-time physical activity versus control, including crossover trials, Outcome- Diastolic blood pressure*

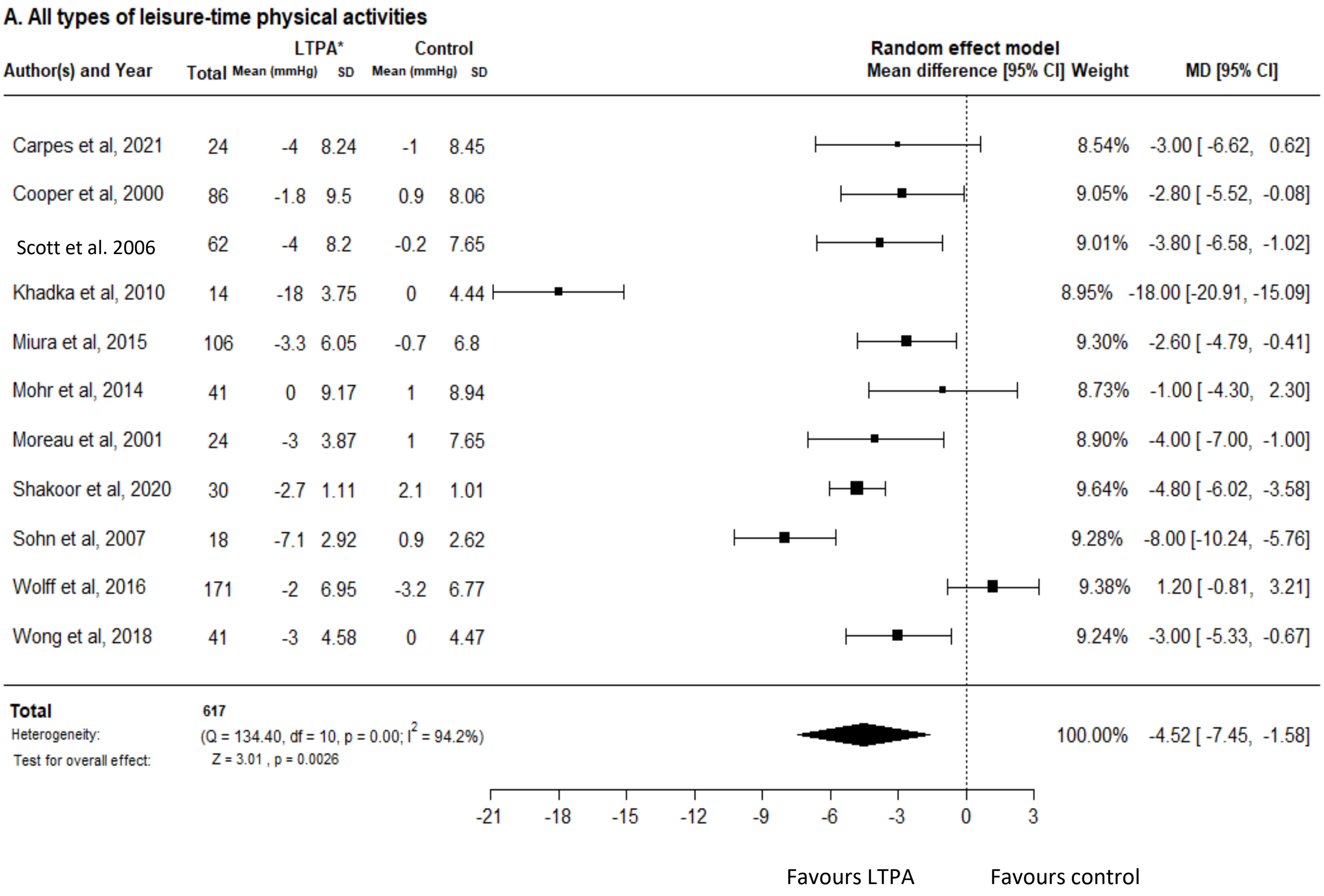

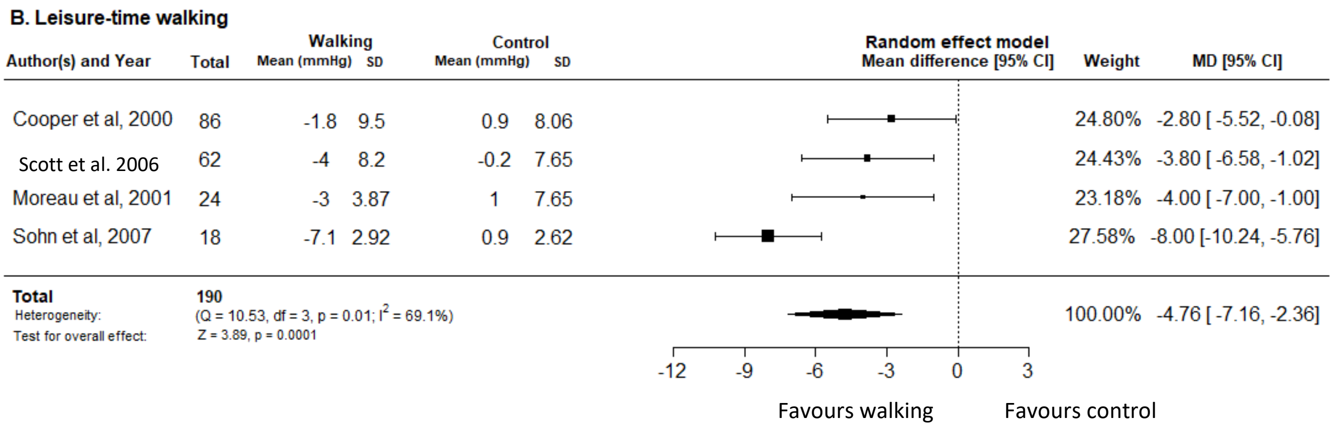

*Carpes et al. 2021 examine the effect of high-intensity exercise, beach tennis on lowering blood pressure.

Appendix: Sensitivity analysis 1: Moderate-intensity leisure-time physical activity versus control including crossover trials, Outcome- Heart best/min

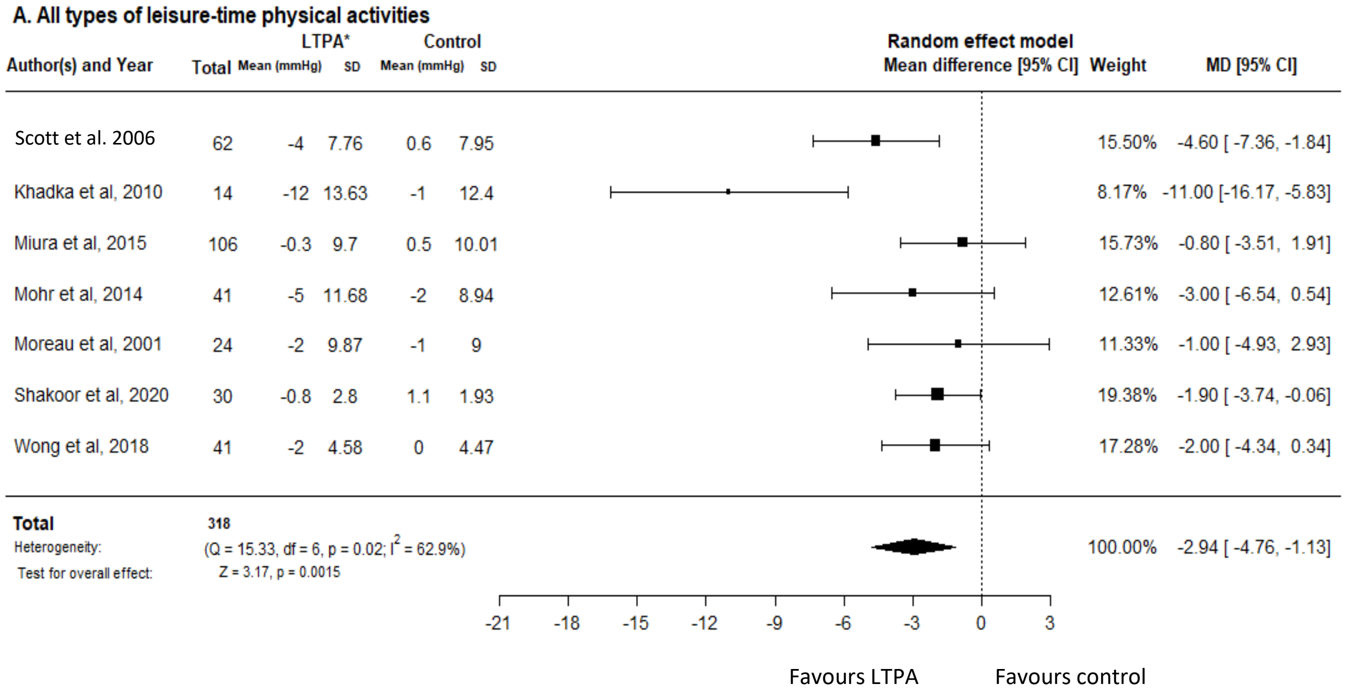

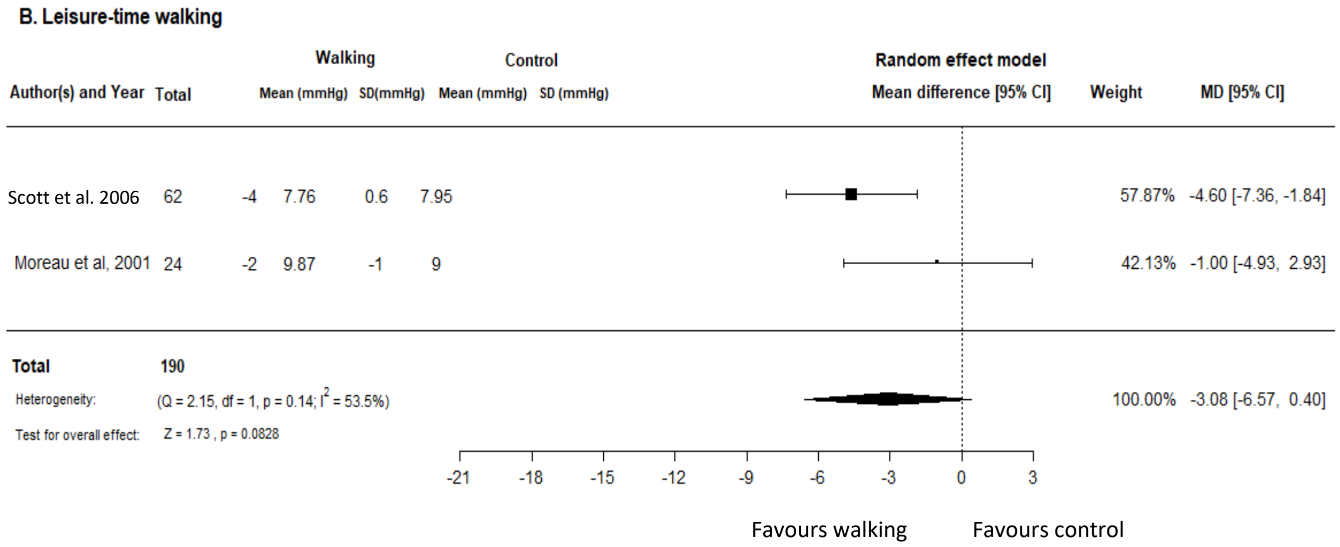

Appendix: Sensitivity analysis 2: Moderate-intensity leisure-time physical activity versus control excluding trials with unclear randomization and treatment concealment, Outcome- Systolic blood pressure

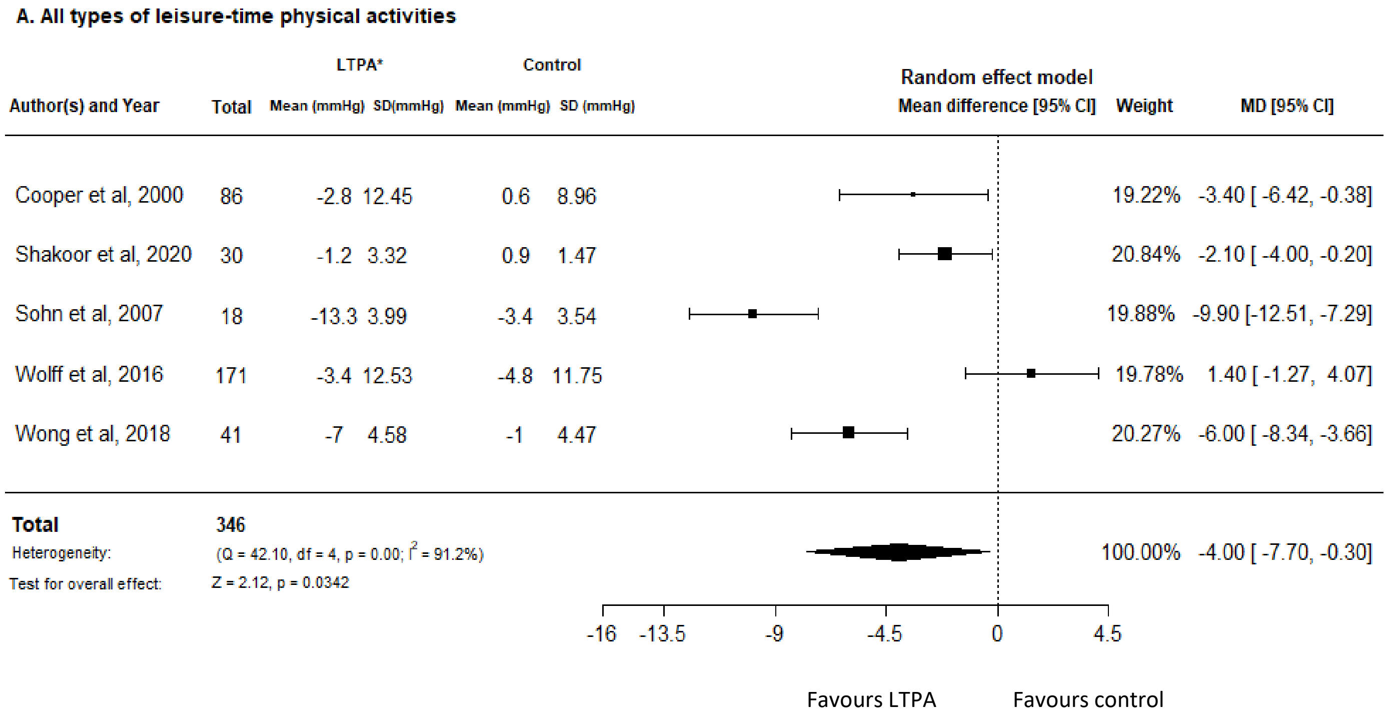

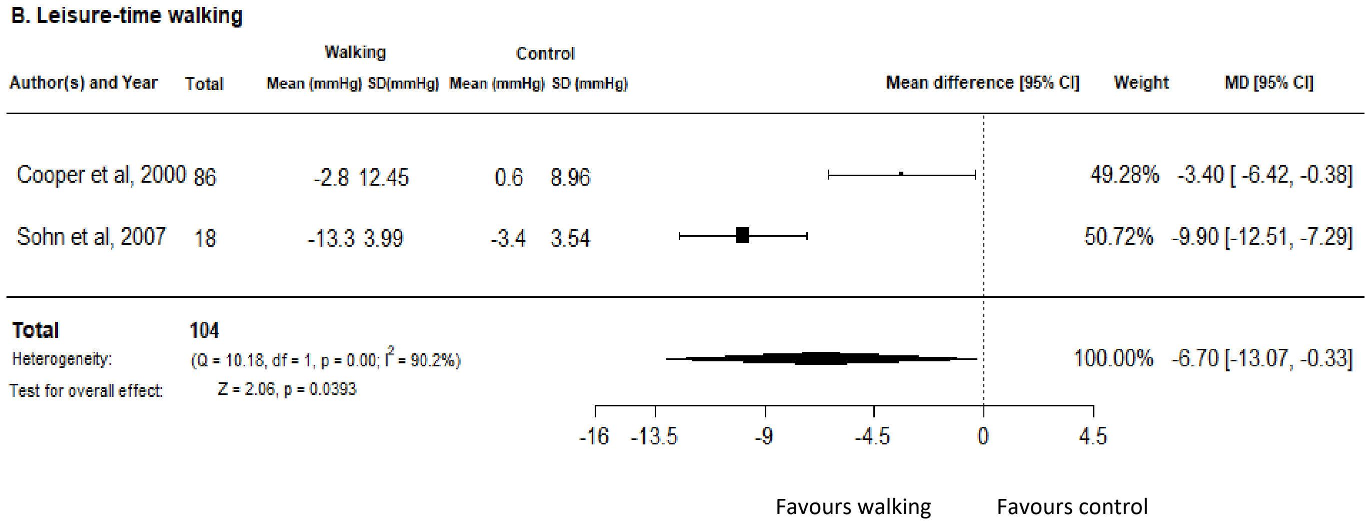

Appendix: Sensitivity analysis 2: Moderate-intensity leisure-time physical activity versus control excluding trials with unclear randomization and treatment concealment, Outcome: Diastolic blood pressure

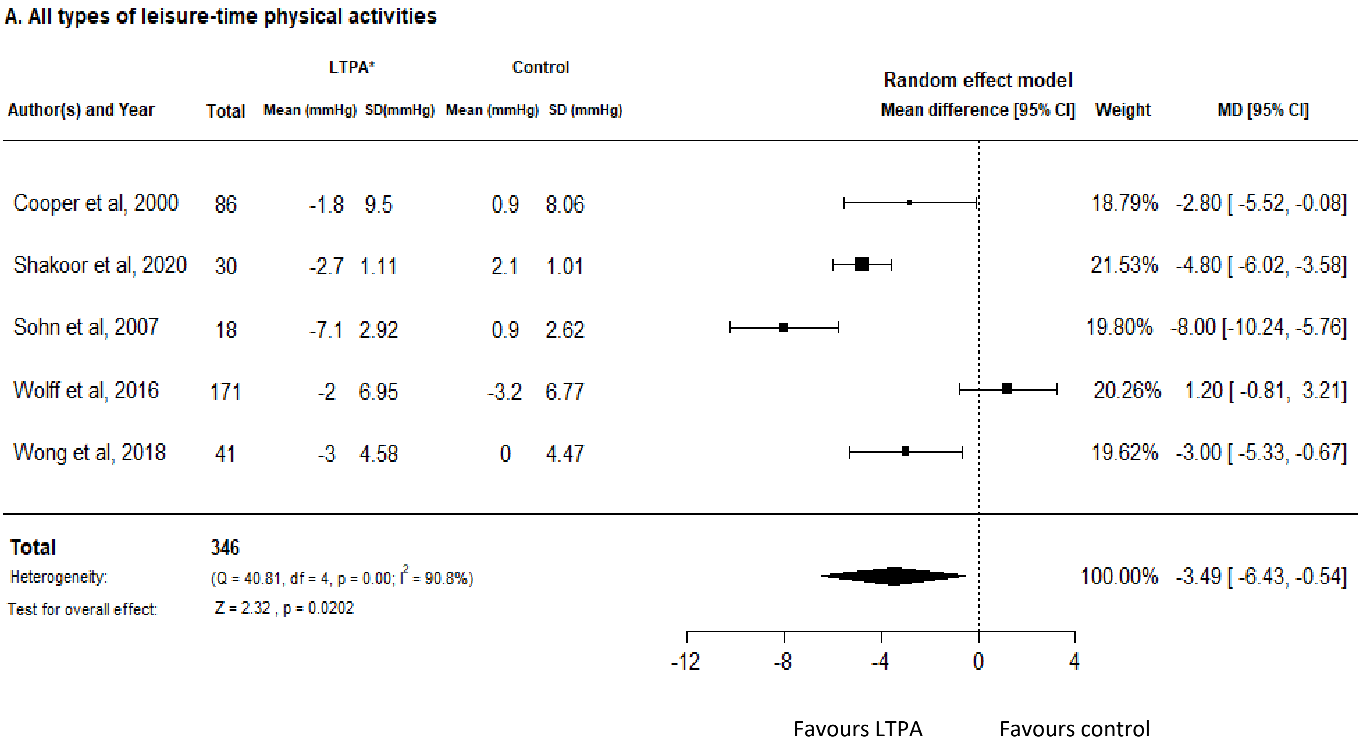

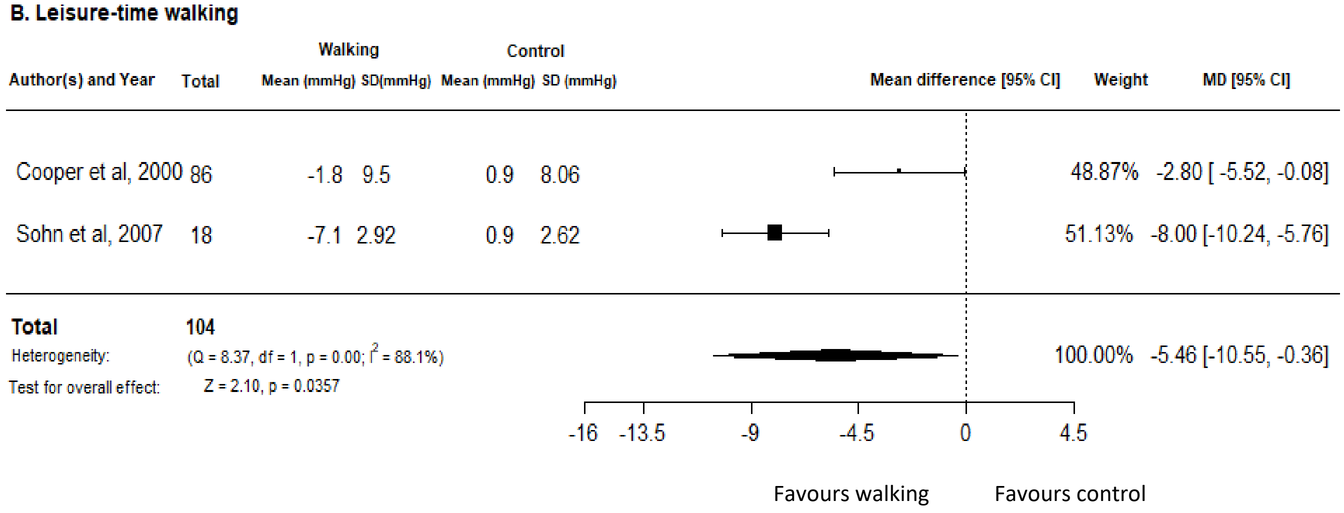

Appendix: Sensitivity analysis 2: Moderate-intensity leisure-time physical activity versus control excluding trials with unclear randomization and treatment concealment, Outcome: Heartbeat/min

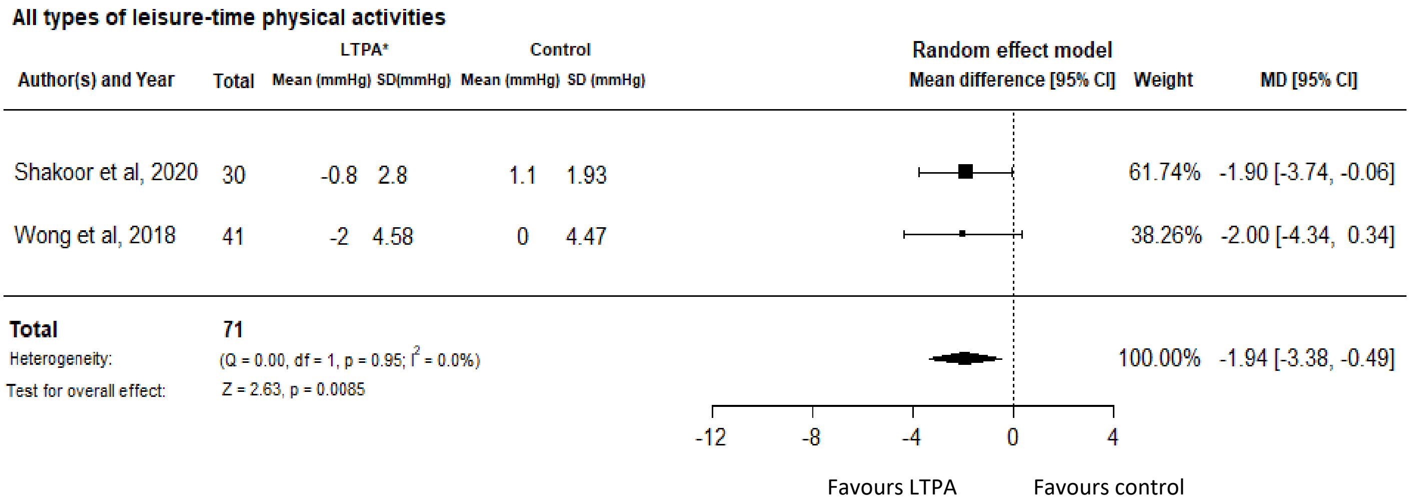

Appendix: Sensitivity analysis 3: Leisure-time physical activity versus control among parallel randomized control trial, Outcome- Systolic blood pressure

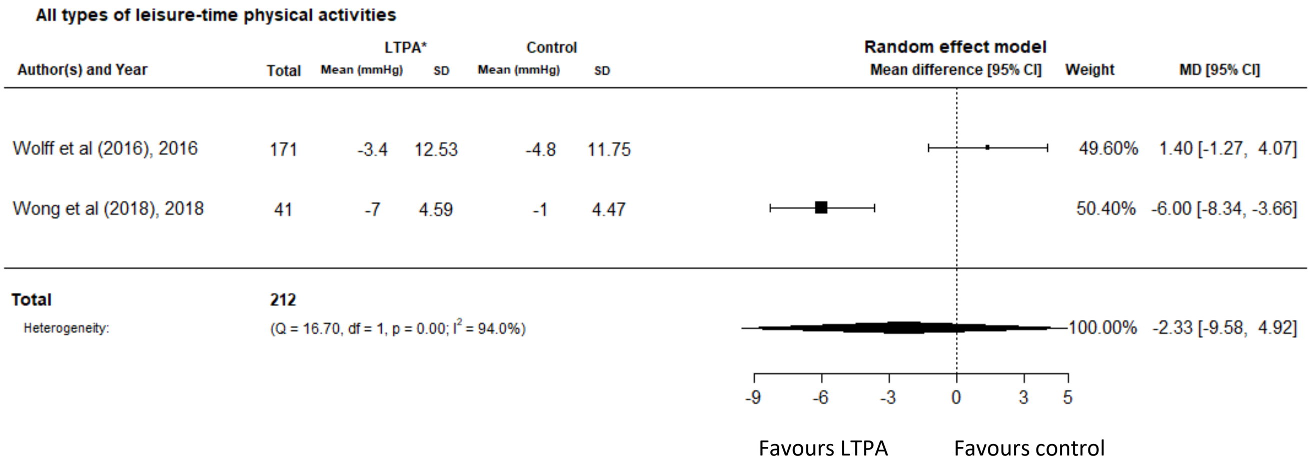

Appendix: Sensitivity analysis 3: Leisure-time physical activity versus control among parallel randomized control trial, Outcome- Diastolic blood pressure

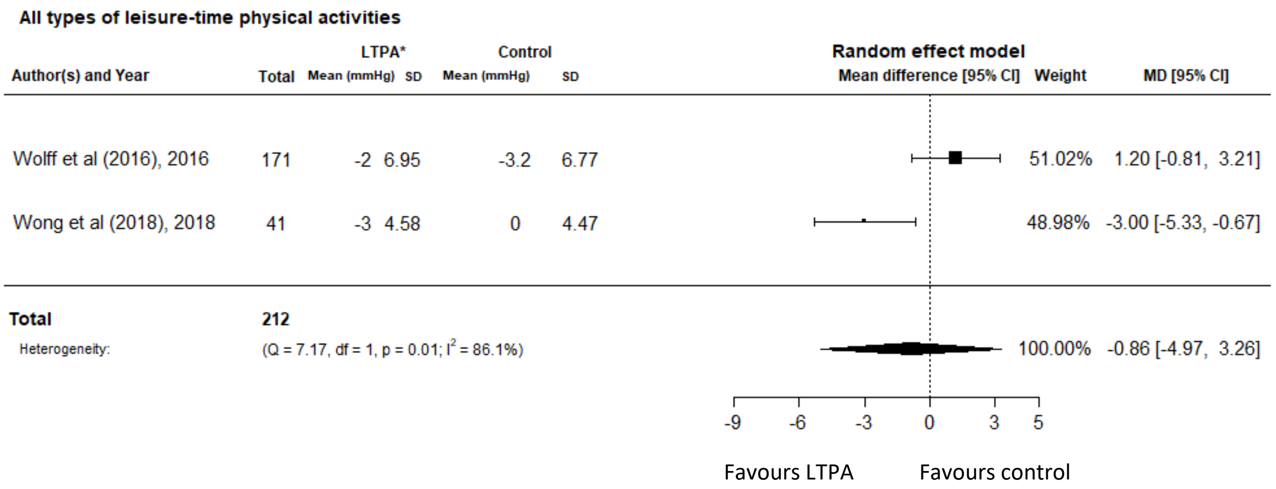

Appendix: Moderate intensity leisure-time physical activities vs non-intervention control, Outcome variable: Heartbeat/min

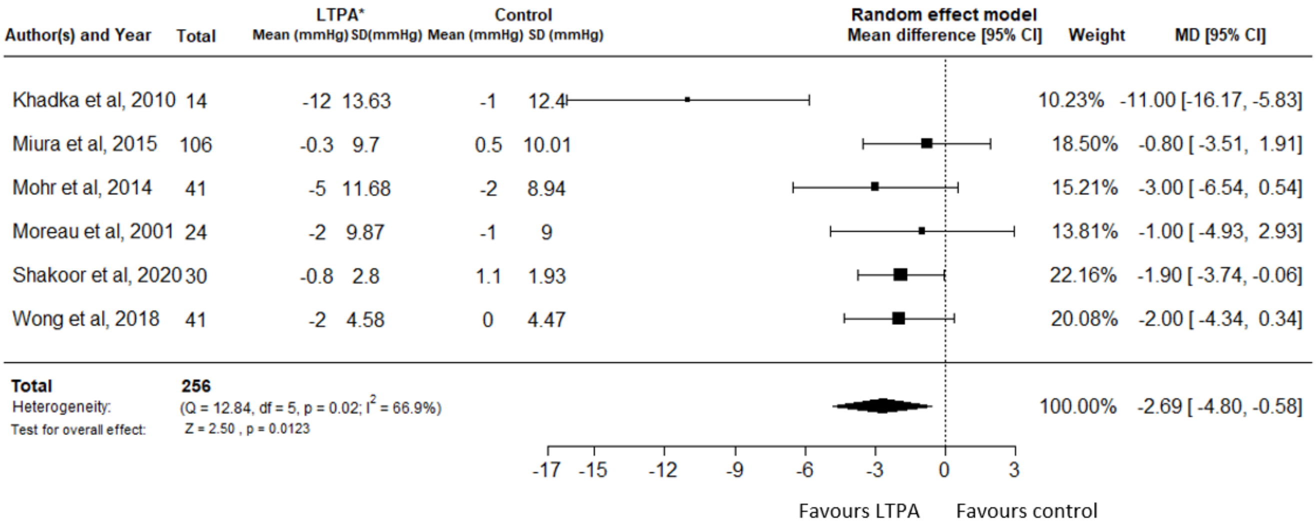

Appendix Table: Evidence profile for moderate-intensity leisure-time physical activity

Certainty assessment							No. of patients	Anticipated absolute effects (95% CI)		Certainty	Importance
№ of studies	Study design	Risk of bias	Inconsistency	Indirectness	Imprecision	Other considerations		Effect with moderate-intensity LTPA	Effect of non-intervention control		
**Systolic blood pressure**											
9	randomised trials	not serious	serious	not serious	serious	none	531	MD 7.6 mm Hg lower (-11.6 to -3.5)	MD 1.9 mm Hg lower (-4.3 to 0.5)	⊕⊕◯◯ Low	Moderate-intensity LTPA may reduce systolic blood pressure
**Diastolic blood pressure**											
9	randomised trials	not serious	serious	not serious	serious	none	531	MD 4.5 mm Hg lower (-8 to -1)	MD 0.2 mm Hg lower (-0.95 to 1.35)	⊕⊕◯◯ Low	Moderate-intensity LTPA may reduce diastolic blood pressure

CI: confidence interval; **MD:** mean difference

Appendix Table: Evidence profile for walking

Certainty assessment							No. of patients	Anticipated absolute effects (95% CI)		Certainty	Importance
№ of studies	Study design	Risk of bias	Inconsistency	Indirectness	Imprecision	Other considerations		Effect with walking	Effect of non-intervention control		
**Systolic blood pressure**											
3	randomised trials	not serious	serious	not serious	serious	none	128	MD 9.0 mm Hg lower (-15.3 to -2.6)	MD 0.77 mm Hg lower (-3.6 to 2.1)	⊕⊕◯◯ Low	Walking may reduce systolic blood pressure
**Diastolic blood pressure**											
3	randomised trials	not serious	serious	not serious	serious	none	128	MD 4.5 mm Hg lower (-8 to -1)	MD 0.2 mm Hg lower (-0.95 to 1.35)	⊕⊕◯◯ Low	Walking may reduce diastolic blood pressure

CI: confidence interval; **MD:** mean difference

Reference

1 Mohr, M. *et al.* High-Intensity intermittent swimming improves cardiovascular health status for women with mild hypertension. *BioMed Research International* **2014** (2014). https://doi.org:10.1155/2014/728289

2 Miura, H., Takahashi, Y., Maki, Y. & Sugino, M. Effects of exercise training on arterial stiffness in older hypertensive females. *European Journal of Applied Physiology* **115** (2015). https://doi.org:10.1007/s00421-015-3168-y

3 Khadka, R., Paudel, B. H., Sharma, V. P., Kumar, S. & Bhattacharya, N. Effect of Yoga on Cardiovascular Autonomic Reactivity in Essential Hypertensive Patients. *Health Renaissance* **8** (2010). https://doi.org:10.3126/hren.v8i2.4421

4 Moreau, K. L. *et al.* Increasing daily walking lowers blood pressure in postmenopausal women. *Medicine and Science in Sports and Exercise* **33** (2001). https://doi.org:10.1097/00005768-200111000-00005

5 Cheung, B. M. Y. *et al.* Randomised controlled trial of qigong in the treatment of mild essential hypertension. *Journal of Human Hypertension* **19** (2005). https://doi.org:10.1038/sj.jhh.1001884

6 Carpes, L., Jacobsen, A., Domingues, L., Jung, N. & Ferrari, R. Recreational beach tennis reduces 24-h blood pressure in adults with hypertension: a randomized crossover trial. *European Journal of Applied Physiology* **121** (2021). https://doi.org:10.1007/s00421-021-04617-4

7 Wolff, M. *et al.* Impact of a short home-based yoga programme on blood pressure in patients with hypertension: A randomized controlled trial in primary care. *Journal of Human Hypertension* **30** (2016). https://doi.org:10.1038/jhh.2015.123

8 Sulaeman, R., Purnamawati, D., Wijayanti, I. G. A. P. W. & Muhasidah. How progressive muscle relaxation exercise affects patient with hypertension? *Rawal Medical Journal* **45** (2020). https://doi.org:10.1097/00004650-200301000-00009

9 Da Nóbrega, T. K. S. *et al.* Walking/running or a recreative soccer game presents similar effectiveness in inducing post-exercise hypotension. *Revista Brasileira de Medicina do Esporte* **19** (2013). https://doi.org:10.1590/S1517-86922013000100006
